# Unraveling the Pharmacological Potential of Lichen Extracts in the Context of Cancer and Inflammation With a Broad Screening Approach

**DOI:** 10.3389/fphar.2020.01322

**Published:** 2020-09-04

**Authors:** Rebecca Ingelfinger, Marina Henke, Luise Roser, Thomas Ulshöfer, Anjuli Calchera, Garima Singh, Michael J. Parnham, Gerd Geisslinger, Robert Fürst, Imke Schmitt, Susanne Schiffmann

**Affiliations:** ^1^ Faculty of Biochemistry, Institute of Pharmaceutical Biology, Chemistry and Pharmacy, Goethe University Frankfurt, Frankfurt, Germany; ^2^ LOEWE Center Translational Biodiversity Genomics, Frankfurt, Germany; ^3^ Branch for Translational Medicine and Pharmacology (TMP), Fraunhofer Institute for Molecular Biology and Applied Ecology IME, Frankfurt, Germany; ^4^ Senckenberg Biodiversity and Climate Research Centre (SBiK-F), Frankfurt, Germany; ^5^ pharmazentrum frankfurt/ZAFES, Institute of Clinical Pharmacology, Goethe University Hospital Frankfurt, Frankfurt, Germany; ^6^ Faculty of Biological Sciences, Institute of Ecology, Evolution and Diversity, Goethe University Frankfurt, Frankfurt, Germany

**Keywords:** lichen extracts, screening, cancer, inflammation, cytotoxicity, migration

## Abstract

Lichen-forming fungi are symbiotic organisms that synthesize unique natural products with potential for new drug leads. Here, we explored the pharmacological activity of six lichen extracts (*Evernia prunastri*, *Pseudevernia furfuracea*, *Umbilicaria pustulata*, *Umbilicaria crustulosa*, *Flavoparmelia caperata*, *Platismatia glauca)* in the context of cancer and inflammation using a comprehensive set of 11 functional and biochemical *in vitro* screening assays. We assayed intracellular Ca^2+^ levels and cell migration. For cancer, we measured tumor cell proliferation, cell cycle distribution and apoptosis, as well as the angiogenesis-associated proliferation of endothelial cells (ECs). Targeting inflammation, we assayed leukocyte adhesion onto ECs, EC adhesion molecule expression, as well as nitric oxide production and prostaglandin (PG)E_2_ synthesis in leukocytes. Remarkably, none of the lichen extracts showed any detrimental influence on the viability of ECs. We showed for the first time that extracts of *F. caperata* induce Ca^2+^ signaling. Furthermore, extracts from *E. prunastri*, *P. furfuracea*, *F. caperata*, and *P. glauca* reduced cell migration. Interestingly, *F. caperata* extracts strongly decreased tumor cell survival. The proliferation of ECs was significantly reduced by *E. prunastri*, *P. furfuracea*, and *F. caperata* extracts. The extracts did not inhibit the activity of inflammatory processes in ECs. However, the pro-inflammatory activation of leukocytes was inhibited by extracts from *E. prunastri*, *P. furfuracea*, *F. caperata*, and *P. glauca*. After revealing the potential biological activities of lichen extracts by an array of screening tests, a correlation analysis was performed to evaluate particular roles of abundant lichen secondary metabolites, such as atranorin, physodic acid, and protocetraric acid as well as usnic acid in various combinations. Overall, some of the lichen extracts tested in this study exhibit significant pharmacological activity in the context of inflammation and/or cancer, indicating that the group lichen-forming fungi includes promising members for further testing.

## Introduction

Lichenized fungi form mutualistic symbioses with photosynthetic microorganisms, usually green algae or cyanobacteria, and additional fungal and bacterial communities. Most species are slow growing and less competitive than other organisms, which is why they have evolved mechanisms to survive in habitats inaccessible to most plants, mosses, or other microorganisms (e.g., arctic tundra, alpine ecosystems, deserts). Secondary metabolites with biological activities protect lichens from being overgrown or eaten (Solhaug et al., 2003; Gauslaa, 2005; Asplund et al., 2015), or from receiving too much UV light (Solhaug et al., 2003). Some of these metabolites might also support the symbiosis itself, by forming water repellent coatings on internal hyphae, which facilitate gas exchange between moist thalli and the environment (Huneck, 2003), or by enabling communication among the different partners in the lichen association. Chemical structures of more than 1,050 different lichen substances have been reported to date (Molnar and Farkas, 2010). Each species is characterized by a specific profile of secondary metabolites (Mitrovic et al., 2011). Most lichen compounds are synthesized by the primary fungal partner in the symbiosis. The main biosynthetic pathways leading to lichen compounds are the shikimic acid pathway (pulvinic acid derivatives), the mevalonic acid pathway (terpenes, carotenoids), and the polyketide pathway (depsides, depsidones, depsones, usnic acids, anthraquinones, xanthones) (Elix and Stocker-Wörgötter, 2008). The highest diversity of compounds is found in the structural classes depsides and depsidones (Huneck and Yoshimura, 1996).

Lichens have been used in traditional systems of medicine since ancient times. Some species, including *Cetraria islandica* and *Evernia prunastri* are useful for treating respiratory diseases (Schöller, 1997), and extracts of *C. islandica* are used as active ingredients in over-the-counter lozenges (“Isla-Moos”). Usnic acid containing species have been used to treat infectious dermatosis and eczema (Schöller, 1997). The varied applications of lichen extracts in medicine include the treatment of skin disorders, wounds, respiratory and digestive issues, as well as obstetric and gynecological problems, and have recently been summarized in a review paper (Crawford, 2019). In this respect, lichen secondary metabolites are increasingly being investigated as potential source of bioactive natural products for pharmaceutical applications (Rankovic and Kosanic, 2014). However, the different studies on the pharmacological potential of lichens have mostly used only one or two bioactivity test systems. The aim of the present study was to apply a broad *in vitro* screening approach in the context of cancer and inflammation. These pathologies represent two major health burdens with an ongoing need for the discovery of new drug leads. We selected 11 *in vitro* screening assays representing functional or biochemical features that are of relevance in these pathologies. The screening assays, their underlying principle, and the cell types used are summarized in **Table 1**. We chose *Evernia prunastri*, *Pseudevernia furfuracea*, *Umbilicaria pustulata*, *Umbilicaria crustulosa*, *Flavoparmelia caperata*, and *Platismatia glauca*, because these species contain typical lichen compounds (depsides, depsidones, usnic acid) in various, partially overlapping, combinations (**Table 2**). Each of the lichen species was extracted with three different solvents representing different degrees of polarity: dichloromethane (D), acetone (A), and methanol 70% (M). Moreover, a correlation analysis was performed to get a hint which of the lichen secondary metabolites might be of importance for the observed effects.

**Table 1:** Summary of the functional and biochemical test systems.

Context	Assay	Principle	Cells
General cell functions	Cell viability	Metabolic activity (CTB assay)	HMECs (human dermal microvascular endothelial cells)
	Intracellular Ca^2+^ levels	Calcium imaging using Fluo-8-AM	HEK293 (human embryonic kidney cells)
	Cell migration	Scratch assay	NIH/3T3 (murine embryonic fibroblasts)
Tumor	Cell viability	Metabolic activity (WST-1 assay)	HCT-116 (human colon carcinoma)
	Apoptosis	Caspase-3 activation, immunocytochemistry	HCT-116
	Cell cycle	Propidium iodide staining, flow cytometry	HCT-116
	Endothelial cell proliferation	Cell counting upon crystal violet staining	HMECs
Inflammation	Leukocyte adhesion	Adhesion of leukocytes onto endothelial cells	THP-1 (human leukemic monocytic cell line) and HMECs
	ICAM-1 expression	Expression of an endothelial cell adhesion molecule	HMECs
	NO levels	Griess assay	RAW 264.7 (murine macrophage cell line)
	PGE_2_ levels	ELISA	RAW 264.7

**Table 2:** Summary of the UV-absorbing natural compounds identified in the lichen extracts by high performance liquid chromatography (HPLC).

	***E. prunastri***			***P. furfuracea***			***U. pustulata***			***U. crustulosa***			***F. caperata***			***P. glauca***			
Extract solvent	A	D	M	A	D	M	A	D	M	A	D	M	A	D	M	A	D	M	
Conprotocetraric acid (β-orcinol depsidone)	–	–	–	–	–	–	–	–	–	–	–	–	1.6	–	0.9	–	–	–	
Unidentified A	–	–	–	–	–	0.8	–	–	–	–	–	–	–	–	–	–	–	–	
Confumarprotocetraric acid (β-orcinol depsidone)	–	–	–	–	–	–	–	–	–	–	–	–	5.0	–	0.8	–	–	–	
Norstictic acid (β-orcinol depsidone)	–	–	–	–	–	–	–	–	–	–	–	–	3.1	–	–	–	–	–	
Protocetraric acid (β-orcinol depsidone)	–	–	–	–	–	–	–	–	–	–	–	–	77.0	21.7	87.6	–	–	–	
Lecanoric acid (orcinol depside, satellite compound of gyrophoric acid)	–	–	–	–	–	–	12.0	–	83.5	3.5	–	6.0	–	–	–	–	–	–	
Unidentified B	–	–	–	–	–	–	–	–	–	–	–	–	1.8	–	–	–	–	–	
Methyl lecanorate (orcinol depside, satellite compound of gyrophoric and crustinic acids)	–	–	–	–	–	–	–	–	–	6.5	–	24.6	–	–	–	–	–	–	
Crustinic acid (orcinol depside)	–	–	–	–	–	–	–	–	–	60.6	–	27.3	–	–	–	–	–	–	
Hiascic acid (orcinol depside)	–	–	–	–	–	–	2.6	–	–	–	–	–	–	–	–	–	–	–	
Evernic acid (orcinol depside)	95.61	80.46	94.14	–	–	–	–	–	–	–	–	–	–	–	–	–	–	–	
Oxyphysodic acid (orcinol depsidone)	–	–	–	17.7	8.6	16.9	–	–	–	–	–	–	–	–	–	–	–	–	
Gyrophoric acid (orcinol depside)	–	–	–	–	–	–	85.4	–	16.5	29.4	–	42.2	–	–	–	–	–	–	
Unidentified C	–	–	–	–	–	0.7	–	–	–	–	–	–	–	–	–	–	–	–	
2’-o-Methylphysodic acid (orcinol depsidone)	–	–	–	4.7	19.8	6.9	–	–	–	–	–	–	–	–	–	–	–	–	
Physodic acid (orcinol depsidone)	–	–	–	72.3	44.9	71.5	–	–	–	–	–	–	–	–	–	–	–	–	
Usnic acid (dibenzofuran derivatives)	3.24	7.02	2.92	–	–	–	–	–	–	–	–	–	10.0	58.9	5.4	–	–	–	
Alectoronic acid (orcinol depsidone)	–	–	–	2.3	–	1.9	–	–	–	–	–	–	–	–	–	–	–	–	
Atranorin (β-orcinol depside)	1.15	6.42	1.73	2.6	19.5	1.3	–	–	–	–	–	–	–	6.5	4.6	22.4	26.8	–	
Unidentified D	–	–	–	–	–	–	–	–	–	–	–	–	–	–	0.8	–	–	–	
Unidentified E	–	–	–	–	–	–	–	–	–	–	–	–	1.6	12.9	–	–	–	–	
Chloroatranorin (β-orcinol depside)	–	6.11	1.20	0.5	7.1	–	–	–	–	–	–	–	–	–	–	77.6	73.2	100.0	

Some of the peaks could not be assigned to any compound and were therefore reported as unidentified. The values are given as percentages. For calculation the area under the peak was used. The peak of the compounds were related to all detected peaks (including peaks which could not be assigned to a compound). The mean of two lichen individuals are depicted.

## Material and Methods

### Cells and Reagents

Fluo-8-AM (ab142773) was purchased from Abcam (Berlin, Germany), ionomycin Ca^2+^-salt, curcumin, staurosporine, and sulfanilamide were purchased from Sigma-Aldrich (Schnellendorf, Germany). CellEvent Caspase-3/7 Green Detection Reagent was purchased from Thermo Fisher Scientific (Frankfurt am Main, Germany). DRAQ5 was purchased from BioLegend (Koblenz, Germany). N-(Naphtyl)ethylendiamindihydrochlorid was purchased from Carl Roth (Karlsruhe, Germany).

### Collection of Lichens

The lichens were collected in Corsica (France). The voucher information and the herbarium accession numbers are documented in **Supplementary Table 1**.

### Lichen Extracts and Characterization of Their Major Secondary Metabolites

We prepared three extracts per lichen individual (two individuals per species) using the following organic solvents: 70% methanol, acetone, and dichloromethane. The extractions were carried out with 30 mg of air-dried lichen thallus and 1.2 ml solvent, at room temperature for 1 h using a tube rotator (Rotator RS-60, lab4you). For cell culture experiments the lyophilizates were dissolved in dimethyl sulfoxide (DMSO) to reach stock solutions with 30 mg/ml. The lichen extracts were further dissolved in media to reach a final concentration of 3 and 30 µg/ml.

Lichen compounds were identified using high performance liquid chromatography (HPLC), based on a protocol by Feige et al. (1993) and described in more detail in Benatti et al. (2013). We used an Agilent 1260 quaternary system with an incorporated degasser and using a Poroshell 120 EC-C18 column (2.7 µm, 3.0 x 50 mm, Agilent) for separation of substances at 30°C. Two solvents are used at a flow rate of 1.4 ml/min. Solvent A contains Aqua Bidest, 30% methanol, and 0.0658% trifluoroacetic acid, and solvent B is 100% methanol. The evaporated extracts were resuspended in 200 µl of methanol and immediately processed for HPLC analysis. One hundred fifty microliters of the methanol extracts were filtered by centrifuging 1 min with 800 rpm through a Pall Acropep Advance Filter Plate. The filtrate is diluted tenfold with methanol and 2 µl is automatically injected after equilibrating the HPLC system for 2 min to solvent A. The run continues isocratically for 0.18 min, solvent B is increased to 58% within 5 min, then up to 100% within the next 5 min, and then for 0.82 min isocratically in 100% B. The run ends with solvent A being increased to back 100% within 0.5 min. The column is flushed for 2 min with 100% solvent B before a new run starts. A diode array detector (DAD) detects compounds at 210, 254, 280, and 310 nm. The spectra (λ = 190–650 nm, 2 nm steps) and retention times of detected peaks are compared against a library of authentic metabolites processed under identical conditions using the OpenLAB CDS ChemStation software of Agilent. We analyzed the relative proportion of the peak area at 254 nm in relation to the total peak area for lichen substances in the species (Serina et al., 1996). The chromatograms are shown in **Supplementary Figure 1**.

### Cell Culture

CDC.EU.HMEC-1, a human microvascular endothelial cell line, was kindly provided by the Centers of Disease Control and Prevention (CDC, Atlanta, USA) (Ades et al., 1992). HMEC-1 cells were cultivated plastic ware coated with collagen G (Biochrom AG, Berlin, Germany) in Endothelial Cell Growth Medium (ECGM, PELOBiotech) containing 10% heat-inactivated fetal calf serum (FCS; Biochrom, Berlin, Germany), 0.25 µg/ml amphotericin B (PAN-Biotech, Aidenbach, Germany), 100 U/ml penicillin (PAN-Biotech), and 100 µg/ml streptomycin (PAN-Biotech).

HEK293 cells [obtained from German Collection of Microorganisms and Cell Cultures GmbH (DSMZ)] were cultured in Dulbecco’s modified Eagle medium (DMEM) (Sigma Aldrich) supplemented with GlutaMAX (Thermo Fisher). NIH3T3 cells (ATCC) were cultured in DMEM GlutaMAX-I Medium (Thermo Fisher) supplemented with 4.5 g/l glucose (Sigma Aldrich) and pyruvate. HCT-116 cells [obtained from DSMZ, Braunschweig, Germany] were cultured in McCoy’s 5A (modified) medium. RAW264.7 (obtained from ATCC) macrophages were cultured in RPMI1640 medium (Sigma Aldrich) (DSMZ). The media for these four cell types contained 10% heat inactivated fetal bovine serum (FBS) and 1% penicillin/streptomycin.

The human monocytic-like cell line THP-1, a cell line derived from the peripheral blood of a 1-year-old boy suffering from acute monocytic leukemia (AML), was purchased from the DSMZ. THP-1 cells were cultured in RPMI-1640 medium (RPMI, PAN-Biotech) containing 10% heat-inactivated FCS, 100 µg/ml streptomycin, and 100 U/ml penicillin (Tsuchiya et al., 1980). All cells were incubated at 37°C in an atmosphere of 5% CO_2_ and 95% air.

### Cell Viability Assays (Metabolic Activity)

A CellTiter-Blue cell viability assay (Promega GmbH, Mannheim, Germany) was performed to analyze the influence of lichen extracts on the metabolic activity of endothelial cells. In brief, confluent HMEC-1 cells were treated with the indicated lichen extracts (3 and 30 µg/ml) or DMSO (0.1%) as vehicle control for 24 h. Four hours before the end of stimulation, CellTiter-Blue reagent containing resazurin was added to the cells in a ratio of 1:10. Resazurin is reduced into fluorescent resorufin by viable cells, which is why the metabolic activity was determined by fluorescence measurements (ex: 579 nm, em: 584 nm) using a microplate reader (Tecan Infinite F200 Pro, Tecan, Männedorf, Switzerland).

20,000 HCT-116 cells were incubated for 24 h at 37°C. Lichen extracts and control (DMSO) were added to the cells and mixed. After 24 h, 10 µl WST-1 reagent (Sigma Aldrich) were added, mixed, and incubated at 37°C for 60 min. Viable cells are characterized by an increase of mitochondrial dehydrogenase activity. Dehydrogenases convert the tetrazolium salt WST-1 to formazan; therefore, the amount of formed formazan dye directly correlates to the number of metabolically active cells in the culture. To detect formazan the absorbance was measured at 440 nm and at 600 nm (reference) using an EnSpire Plate Reader (PerkinElmer, Waltham, MA, USA). The absorbance at 440 nm was normalized to the absorbance at 600 nm. The sample values were corrected with the background wells (wells with medium and without cells).

### Analysis of Intracellular Ca^2+^ Levels

50,000 HEK293 cells were seeded in a 96-well poly-D-lysine plate and incubated at 37°C for 24 h. Cells were incubated with 4 µM Fluo-8-AM in Hanks’ Balanced Salt Solution (HBSS) for 1 h at 37°C. DMSO was used as negative control. After 1 h, the Fluo-8/HBSS was replaced by 100 µl HBSS. Five images per second were taken using an ImageXpress Micro Confocal High Content Imaging System (Molecular Device, San Jose, USA). Then, lichen extracts (3 and 30 µg/ml), DMSO (negative control), or 5 µM ionomycin (Sigma Aldrich; positive control) were added to the cells. For the next 20 s, an image was taken every second. The data were analyzed with the MetaXpress Software. A threshold of fluorescence intensity was defined using cells before treatment, and all cells with a fluorescence signal above the threshold level were counted. The number of these cells in the lichen extract treated samples were related to the cells in the ionomycin-treated sample.

### Migration Assay

120,000 NIH3T3 cells were plated in a clear poly-D-lysine-coated 24-well plate and incubated for 24 h at 37°C in presence of lichen extracts (30 µg/ml) or DMSO. A scratch was made in the cell layer using a 10 µl pipette tip. Cells were washed once with medium containing 2% FBS and 25 mM 4-(2-hydroxyethyl)-1-piperazineethanesulfonic acid (HEPES) to remove non-adherent cells or debris. Wells were filled with 500 µl medium containing 2% FBS, 25 mM HEPES, and lichen extracts (30 µg/ml) or DMSO or 625 nM cytochalasin D (Sigma Aldrich). The 24-well plate was covered with an adhesive foil and placed for 24 h in the ImageXpress and incubated at 37°C for 24 h. Every 90 min an image from the scratch was taken by the ImageXpress device (10x magnification). For analysis the length of the scratch was measured using MetaXpress6 Software. The length of the scratch was plotted against the time. The IC_50_ was determined. The IC_50_ defines the time which is needed to close 50% of the scratch.

### Apoptosis Assay (Caspase-3 Activation)

20,000 HCT 116 cells were seeded in a black poly-D-lysine-coated 96-well plate and incubated for 24 h at 37°C. The culture medium was replaced by 100 µl DMEM-medium without phenol red (Gibco, 21063-029) supplemented with 10% FBS and 1% penicillin/streptomycin. Lichen extracts or control (DMSO) were added to the cells and incubated for 24 h at 37°C. One microliter of CellEvent Caspase-3/7 Green Detection Reagent (1:10 diluted in DMEM-medium without phenol red) was added and incubated for 90 min at 37°C (without CO_2_). Afterwards, 1 µl of DRAQ5 (1:25 diluted in DMEM-medium without phenol red) was added and the cells were incubated for 30 min at room temperature. An image was taken using the ImageXpress Micro Confocal High Content Imaging System (Molecular Device, San Jose, USA). The fluorescence signal of cell nuclei was detected in the Cy5 channel (red), the signal of apoptotic cells in the fluorescein isothiocyanate (FITC) channel (green). The percentage of dead cells was determined using the “live/dead” analysis tool from Molecular Device by calculating the ratio of apoptotic cells to all cells.

### Cell Cycle Analysis

20,000 HCT116 were seeded in a 96-well cell culture plate and cultured for 24 h at 37°C. Then, cells were treated with the different lichen extracts (3 and 30 µg/ml) or control substances for 24 h. As control substances, 20 µM curcumin and 0.2 µM staurosporine were used to induce a G2- and an S-block, respectively. Untreated cells were used as negative control. A G1/S-block was induced by medium without 10% FBS. Cells were harvested, suspended in 200 µl sample buffer [1 g/l glucose in phosphate-buffered saline (PBS) without Ca^2+^ and Mg^2+^], mixed, centrifuged (200 g, 4 min, 4°C) and supernatant was discarded. This step was repeated once. The cells were fixed with 150 µl of ice-cold 70% ethanol overnight (>18 h) at 4°C. The cell pellet was washed with sample buffer, resuspended in 100 µl staining buffer (20 µg/ml propidium iodide and 0.2 mg/ml RNase in sample buffer), and incubated for 40 min at room temperature. Samples were measured within 24 h in a MACSQuant Analyzer (Miltenyi Biotec GmbH, Bergisch Gladbach, Germany). Cell cycle distribution was determined using FlowJo software.

### Proliferation Assay

A staining with crystal violet solution was used to investigate the influence of lichen extracts on the proliferation of endothelial cells. Therefore, HMEC-1 cells (2 x 10^3^ cells per well) were seeded in 96-well plates. After 24 h the cells were treated with the indicated lichen extracts (3 and 30 µg/ml) or DMSO (0.1%) as vehicle control for 72 h. The cells were washed once with PBS containing Ca^2+^ and Mg^2+^ before they were fixed with a methanol-ethanol solution (ratio 2:1) for 10 min and stained with crystal violet solution (0.5% in 20% methanol, Sigma-Aldrich, Taufkirchen, Germany) for 15 min. The unbound residual crystal violet solution was removed with tab water and the cells were dried for 15 min at 37°C in a drying cabinet before crystal violet bound to DNA was resolved in 20% acetic acid. The absorption of reconstituted crystal violet was measured at 590 nm using a microplate reader (Tecan). The relative proliferation rate of HMEC-1 cells was calculated on the basis of data from untreated control cells, which were fixed and stained directly 24 h after seeding.

### Analysis of the Adhesion of Leukocytes to Endothelial Cells

The impact of lichen extracts on the adhesion of the human monocytic-like cell line THP-1 onto endothelial cells was analyzed in cell adhesion assays, in which untreated THP-1 cells were allowed to adhere to HMEC-1 cells treated with lichen extracts. In brief, confluent HMEC-1 cells were pre-treated with the indicated lichen extracts (3 and 30 µg/ml) or DMSO (0.1%) as vehicle control for 30 min and activated with TNF (10 ng/ml) for 24 h. CellTracker Green (5 µM, Thermo Fisher Scientific, Waltham, MA, USA) was used to stain THP-1 cells in serum-free RPMI for 30 min at 37°C in a water bath. The THP-1 cells were then washed once with serum-free RPMI and the cell concentration was adjusted with RPMI to 2 x 10^5^ cells per ml. THP-1 cells (2 x 10^4^ cells per well) were allowed to adhere for 5 min before non-adherent cells were removed by washing with PBS containing Ca^2+^ and Mg^2+^. The adhesion of THP-1 cells was quantified by fluorescence measurements (ex: 485 nm, em: 535 nm) using a microplate reader (Tecan). Relative adhesion levels were calculated by normalization to the number of HMEC-1 cells that were present after treatment with lichen extracts in combination with TNF, which was determined by staining with crystal violet as described above.

### Cell Surface Expression of ICAM-1

The effect of lichen extracts on the expression of the intercellular adhesion molecule 1 (ICAM-1) on the cell surface of endothelial cells was investigated using a fluorescence-labeled antibody: confluent HMEC-1 cells were pre-treated with the indicated lichen extracts (3 and 30 µg/ml) or DMSO (0.%) as vehicle control for 30 min and activated with TNF (10 ng/ml) for 24 h. The cells were washed once with PBS before they were incubated for 45 min on ice with a FITC-labeled ICAM-1 antibody (1%, Bio-Rad Laboratories, Munich, Germany) in Medium 199 (Sigma-Aldrich) containing 0.2% bovine serum albumin (BSA, Sigma-Aldrich), 100 U/ml penicillin, and 100 µg/ml streptomycin. Afterwards, unbound antibody was removed by washing with PBS containing Ca^2+^ and Mg^2+^ and the cell surface expression of ICAM-1 was quantified by fluorescence measurements (ex: 485 nm, em: 535 nm) using a microplate reader (Tecan). As with the cell adhesion assay, the relative expression levels were calculated by normalization to the number of HMEC-1 cells that were present after treatment with lichen extracts in combination with TNF, which was determined by staining with crystal violet as described above.

### Measurements of Nitric Oxide and PGE_2_ Levels

20,000 RAW264.7 macrophages per well were plated in a 96-well cell culture plate and cultured for 24 h at 37°C. To test if the lichen extracts induce nitric oxide (NO) or prostaglandin E2 (PGE_2_) synthesis, the lichen extracts, control (DMSO), and positive control (100 ng/ml lipopolysaccharide; LPS) were added. To test if the lichen extracts inhibit NO or PGE_2_ synthesis, the cells were pre-incubated with lichen extracts or control (DMSO) for 30 min before 100 ng/ml LPS were added. After 24 h supernatants were collected and stored at −80°C.

NO was determined with the Griess method. Briefly, for the standard curve, different concentrations of sodium nitrite (0–50 µM) were prepared in medium. Eighty microliters of cell supernatant or standard sample were added to a 96 well microplate and thereafter, 20 µl sulfanilamide solution (120 mg sulfanilamide in 30 ml 1 N hydrochloric acid) und 20 µl naphthylenediamine solution (180 mg N-(1-naphthyl)ethylenediamine dihydrochloride in 30 ml water) were added. After 15 min, the absorbance (540 nm) was measured EnSpire Plate Reader (PerkinElmer, Waltham, MA, USA).

PGE_2_ was determined using a competitive ELISA from Enzo Life Sciences. The ELISA was performed according to the manufactures protocol. Briefly, anti-PGE_2_ is bound on 96-well plates. Test samples and alkaline phosphatase conjugated-PGE_2_ were added to the wells. After incubation the excess reagents were washed away and p-nitrophenol phosphate was added and catalyzed by alkaline phosphatase to produce p-nitrophenol a yellow colored product which is detected with EnSpire Plate Reader (PerkinElmer, Waltham, MA, USA). The intensity of the yellow coloration is inversely proportional to the amount of PGE_2._

### Correlation Analysis

Correlation analysis of lichen substances and assay outcomes were conducted with R (V3.6.1; available online https://www.R-project.org/) (Macdonald et al., 1999). Data was loaded in the R workspace and tested for normality (Shapiro-Wilk Test). A Spearman correlation matrix as well as the exact p-values were calculated (R package v4.3-0 available online https://CRAN.R-project.org/package=Hmisc). Briefly, the correlation analysis relies on Spearman’s rho. The data considered for the correlation are the treatment (only lichen species) and the solvent. As only the highest dose was considered (30 µg/ml), the concentration presents no third factor. Furthermore, interactions were not included in the analysis. Analysis was performed with the averaged result for the 30 µg/ml assay outcome (mean of the biological replicates) and the HPLC analysis (mean of the biological replicates). The peak area of a specific natural compound were related to the total peak area of all lichen substances in the species. The HPLC data were classified in four groups: 0 (natural compound not detectable), 1 (amount of natural compounds from 0.01 to 33%), 2 (amount of natural compound from 34 to 66%), and 3 (amount of natural compound from 67 to 100%). For this type of experiment, no correction for multiple testing was performed. The correlation matrix was visualized depicting the correlation coefficient r and the significance levels (*p ≤ 0.05) (visualization of a Correlation Matrix (V0.84) available online https://github.com/taiyun/corrplot; ColorBrewer Palettes (V1.1-2) available online https://CRAN.R-project.org/package=RColorBrewer).

### Statistics

Each result is presented as mean ± standard error of the mean (SEM). The experiments were performed in technical triplicates. The number of biological replicates is indicated in the figure legends (n). All data were analyzed with two-way ANOVA and Bonferroni’s multiple comparison tests except of the data from the cell cycle analysis that were analyzed by the Dunnett’s multiple comparison tests. For all calculations and creation of graphs, GraphPad Prism 7 was used and p ≤ 0.05 was considered as the threshold for significance.

## Results

### The Influence of Lichen Extracts on General Cell Functions

**Figure 1:**
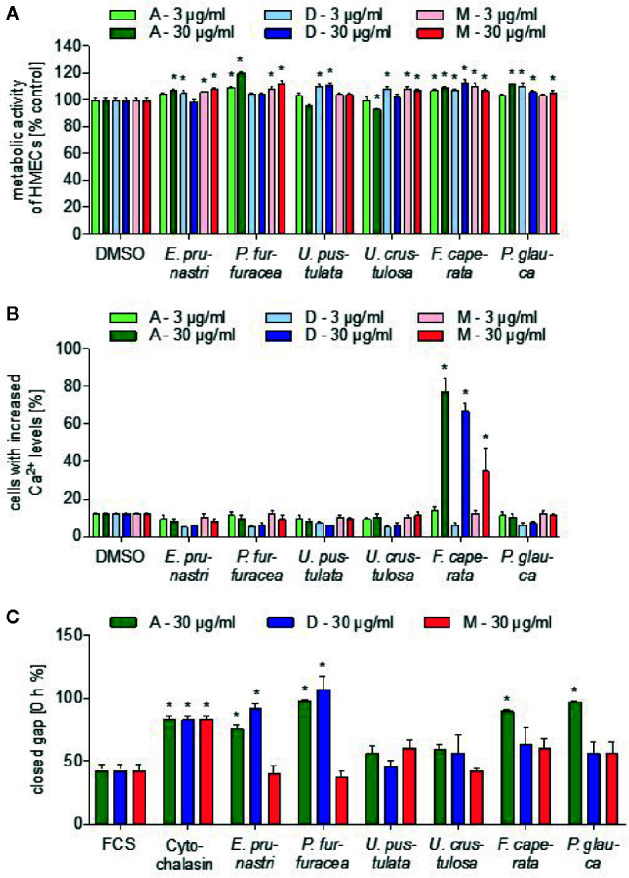
Effects of lichen extracts on general cell functions. **(A)** For the cell viability assay, HMEC-1 cells were grown to confluence and treated with lichen extracts (3 and 30 µg/ml) or dimethyl sulfoxide (DMSO) (control) for 24 h. Resazurin-containing CellTiter-Blue reagent was added for the last 4 h of treatment. The metabolic activity was quantified by fluorescence measurements of resorufin. Data are expressed as mean ± SEM. n=2, *p ≤ 0.05 *versus* control. **(B)** For the Ca^2+^-assay, HEK293 cells were preincubated with Fluo-8-AM. Lichen extracts (3 and 30 µg/ml) or DMSO (control) were added for 5 min. Data are expressed as mean ± SEM. n=3, *p ≤ 0.05 *versus* control. **(C)** For the scratch assay, the NIH3T3 monolayer was scraped in a straight line and thereafter treated with 30 µg/ml of lichen extracts or with 650 nM cytochalasin (positive control) or DMSO (control) for 24 h. The size of the gap after 12.5 h was related to the size of the gap at 0 h and shown as % value. Data are expressed as mean ± SEM. n=2, *p ≤ 0.05 *versus* control.

First, we studied the action of the lichen extracts on the viability of human non-cancer cells. In particular, a monolayer of human endothelial cells (ECs) was used to study the influence of the lichen extracts on metabolic activity (CellTiter-Blue assay). As shown in **Figure 1A**, none of the lichen extracts (3 and 30 µg/ml, 24 h) affected the metabolic activity of ECs in a biologically relevant manner.

Since calcium ions (Ca^2+^) are an important second messenger in cancer and inflammation (Cui et al., 2017), we investigated if lichen extracts influence cellular Ca^2+^ levels in HEK293 cells by using the cell-permeable calcium-sensitive dye Fluo-8-AM. Interestingly, all extracts of *F. caperata* (30 µg/ml) triggered a strong increase in the cellular Ca^2+^ levels (**Figure 1B**), whereas no other lichen extract showed an influence in this assay system.

Cell migration is an important function of tumor cells specifically in the metastatic process and of leukocytes during inflammatory processes (Wirtz et al., 2011; Trepat et al., 2012). To test the influence of the lichen extracts on the migratory capacity of cells, a scratch assay was performed with a mouse embryonic fibroblast (NIH/3T3 cells) monolayer. Lichen extracts and a positive control (625 nM cytochalasin D, an inhibitor of actin polymerization) were added and the cell migration into the cell-free area was followed by live-cell imaging. The distance of the gap after 12 h was related to the distance of the initial gap. As shown in **Figure 1C** *P. furfuracea* and *E. prunastri* (30 µg/ml; dichloromethane and acetone extract) strongly prolonged the time to close the gap. Interestingly, also the acetone extract from *P. caperata* and *P. glauca* prevented the closing of the gap.

### Lichen Extracts Exert Anti-Tumor Activity

**Figure 2:**
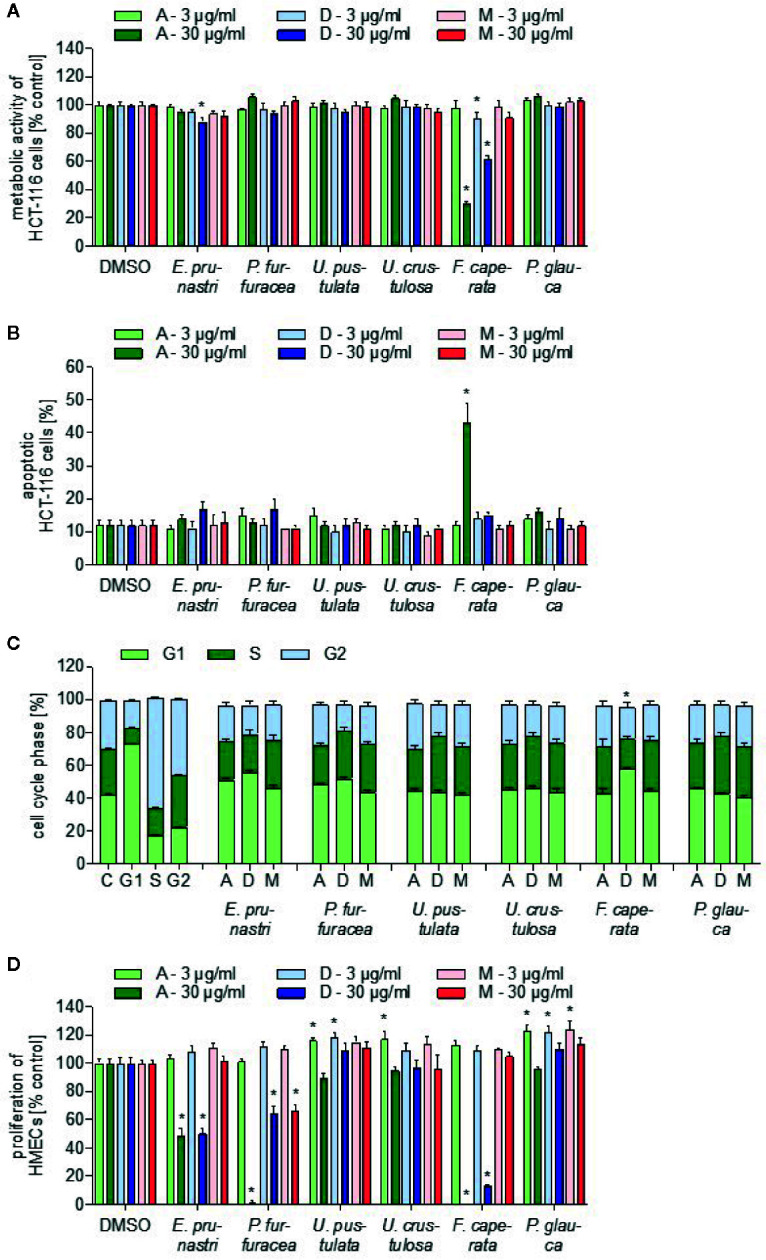
Toxicity characterization of lichen extracts. **(A)** For the cell viability assay, HCT-116 cells were incubated with 3 or 30 µg/ml lichen extracts or dimethyl sulfoxide (DMSO) (control) for 24 h. WST-1 dye was added for 60 min and the viability was quantified by detection of formazan. Data are expressed as mean ± SEM. n=2, *p ≤ 0.05 *versus* control. **(B)** For the apoptosis assay, HCT-116 cells were incubated with 3 and 30 µg/ml lichen extracts or DMSO (control) for 24 h. Apoptotic cells were stained with a caspase-3-detecting antibody. Data are expressed as mean ± SEM. n=2, *p ≤ 0.05 *versus* control. **(C)** For the cell cycle analysis, HCT-116 cells were incubated for 24 h with 30 µg/ml lichen extracts or DMSO (control). Cells were stained with propidium iodide and measured using flow cytometry. Data are expressed as mean ± SEM. n=3, *p ≤ 0.05 *versus* control. Significances are shown when all three cell cycle phases were significantly different. **(D)** For the proliferation assay, HMEC-1 cells were grown in low-density and treated after 24 h with lichen extracts (3 and 30 µg/ml) for 72 h. Cells were stained with crystal violet solution. The amount of DNA-bound crystal violet was detected by absorbance measurements. Data are expressed as mean ± SEM. n=2, *p ≤ 0.05 *versus* control.

The lichen extracts were analyzed for their ability to influence the survival of tumor cells by three different assay systems that measure the metabolic activity of tumor cells, the rate of apoptosis, and cell cycle distribution. The metabolic cell activity was detected with a WST-1 assay using HCT-116 cells. As shown in **Figure 2A**, only extracts of *F. caperata* (30 µg/ml, 24 h) reduced the viability of cancer cells in a biologically relevant manner: the acetone extract reduced cell viability by about 70%, and the dichloromethane extract by approx. 40%. Since caspase-3 activation is a crucial component of the apoptotic machinery (Porter and Janicke, 1999), the influence of the different lichen extracts on tumor cell apoptosis was analyzed by measuring caspase-3 activation in HCT-116 cells. Interestingly, only the acetone extract (30 µg/ml, 24 h) of *F. caperata* increased the basal rate of apoptosis (**Figure 2B**).

The distribution of cells in the different cell cycle phases was analyzed by measuring the content of propidium iodide-stained DNA in HCT-116 cells using flow cytometry. As positive control, a G1-, S-, and G2-block was induced by starvation (medium without FBS), 0.2 µM staurosporine, and 20 µM curcumin, respectively. Only 30 µg/ml (24 h) of the *F. caperata* dichloromethane extract led to a significant increase in the percentage of cells in the G1 phase and to a significant decrease of cells in the S phase (**Figure 2C**, **Supplemental Figure 2**).

The proliferation of ECs is a hallmark of angiogenesis—the outgrowth of new blood vessels of existing ones—which is of great importance during solid tumor growth. Some of the extracts strongly inhibited the proliferation of ECs: as shown in **Figure 2D**, the acetone and dichloromethane extracts of *E. prunastri*, *P. furfuracea*, and *F. caperata* (30 µg/ml, 72 h) reduced EC proliferation by at least 40%. In addition, the methanol extract of *P. furfuracea* (30 µg/ml) also inhibited EC proliferation by about 40%.

### Effects of Lichen Extracts on the Pro-Inflammatory Activation of Endothelial Cells and Leukocytes

**Figure 3:**
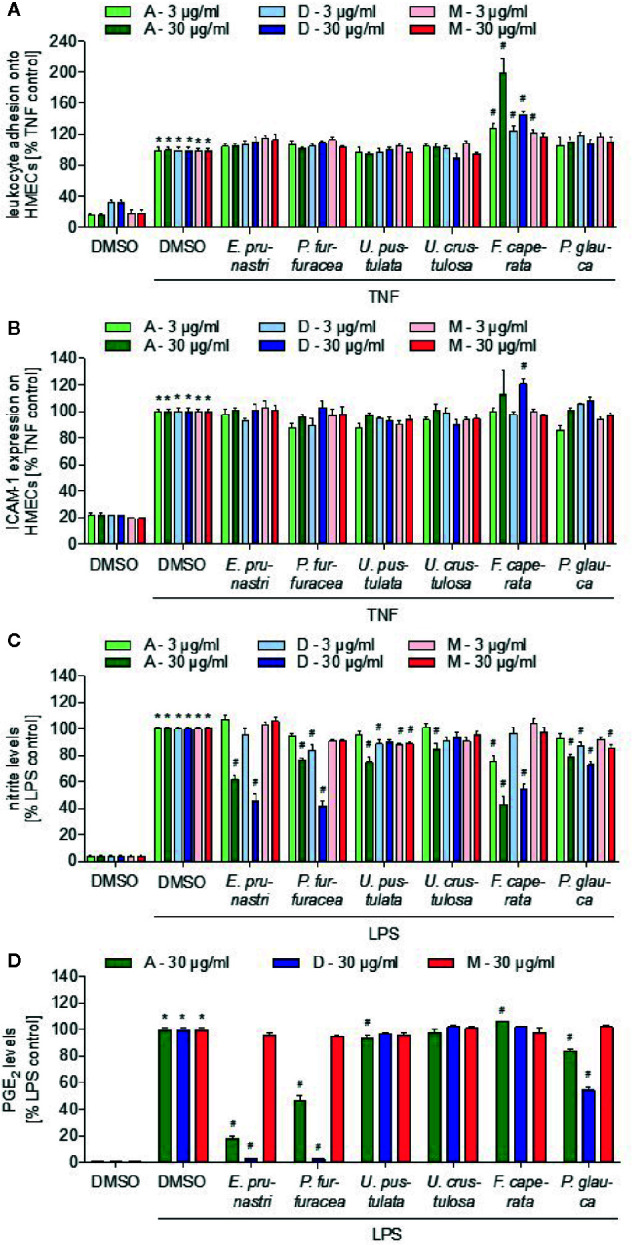
Effects of lichen extracts on cellular functions. **(A, B)** HMEC-1 cells were grown to confluence, preincubated with lichen extracts (3 and 30 µg/ml) for 30 min, and activated with TNF (10 ng/ml) for 24 h. **(A)** For the leukocyte adhesion assay, untreated THP-1 cells were stained with CellTracker Green and were allowed to adhere onto the treated endothelial cells (ECs) for 5 min. Non-adherent THP-1 cells were removed by washing. The adhesion of leukocytes onto ECs was quantified by fluorescence measurements. **(B)** For ICAM-1 expression analysis, cells were incubated with a fluorescein isothiocyanate (FITC)-labeled ICAM-1 antibody for 45 min and the cell surface expression of ICAM-1 was quantified by fluorescence measurements after washing. **(A, B)** Data are expressed as mean ± SEM. n=2, *p ≤ 0.05 *versus* dimethyl sulfoxide (DMSO) control; ^#^p ≤ 0.05 TNF control. **(C, D)** For the nitric oxide (NO) **(C)** and prostaglandin E2 (PGE_2_) **(D)** inhibition assay, RAW macrophages were preincubated with 3 or 30 µg/ml lichen extracts or DMSO (control) for 30 min followed by the addition of 100 ng/ml lipopolysaccharide (LPS) for 24 h. Data are expressed as mean ± SEM. n=3, *p ≤ 0.05 *versus* DMSO control; ^#^p ≤ 0.05 LPS control.

Inflammatory processes are associated with the presence of leukocytes in the inflamed tissue. For the extravasation of leukocytes from the blood into the tissue, the adhesion of the white blood cells onto the vascular endothelium is a prerequisite. Using the monocytic cell line THP-1, we screened the lichen extracts for their ability to reduce the adhesion of THP-1 cells onto a TNF-activated EC monolayer. Besides the *F. caperata* acetone extract (30 µg/ml, 24 h), which surprisingly increased the adhesion of THP-1 cells onto TNF-activated ECs, none of the extracts inhibited the adhesion process (**Figure 3A**). In a second assay system, we screened for an inhibition of the TNF-induced expression of the cell adhesion molecule ICAM-1, which is crucially involved in the adhesion of leukocytes onto ECs. None of the extracts exerted an inhibitory activity on the ICAM-1 expression (**Figure 3B**). Since only ECs were treated with the lichen extracts, we can conclude that inflammation-associated processes in the vascular endothelium are not addressed by the lichen extracts.

Furthermore, we were interested in the action of the lichen extracts on the pro-inflammatory activation of leukocytes. We investigated if the lichen extracts influence the production of two major inflammatory mediators produced by leukocytes, nitric oxide (NO), and prostaglandin E_2_ (PGE_2_). Murine macrophages (RAW264.7) were treated with lipopolysaccharide (LPS) to induce the release of NO and PGE_2_ and with the lichen extracts for 24 h. The concentration of NO and PGE_2_ was determined in the cell culture supernatant. The acetone extract (30 µg/ml) of *E. prunastri* and *F. caperata* strongly reduced the levels of NO (**Figure 3C**), while weak effects were observed for *P. furfuracea*, *U. pustulata*, and *P. glauca* extracts. The dichloromethane extracts (30 µg/ml) of *E. prunastri*, *P. furfuracea*, and *F. caperata* showed a strong inhibition of NO formation, whereas the *P. glauca* dichloromethane extract had weaker effects. The acetone extract of *E. prunastri* and *P. furfuracea* (30 µg/ml) strongly reduced the levels of PGE_2_ (**Figure 3D**), while weak effects were observed for *P. glauca* extracts. In addition, the dichloromethane extracts (30 µg/ml) of *E. prunastri*, *P. furfuracea*, and *P. glauca* strongly inhibited PGE_2_ formation. Of note, none of the lichen extracts affected the basal release of NO or PGE_2_ form quiescent (i.e., not LPS-activated) RAW cells in a biologically relevant manner (**Supplemental Figures 3** and **4**).

### Natural Compounds Identified in the Lichen Extracts

**Figure 4:**
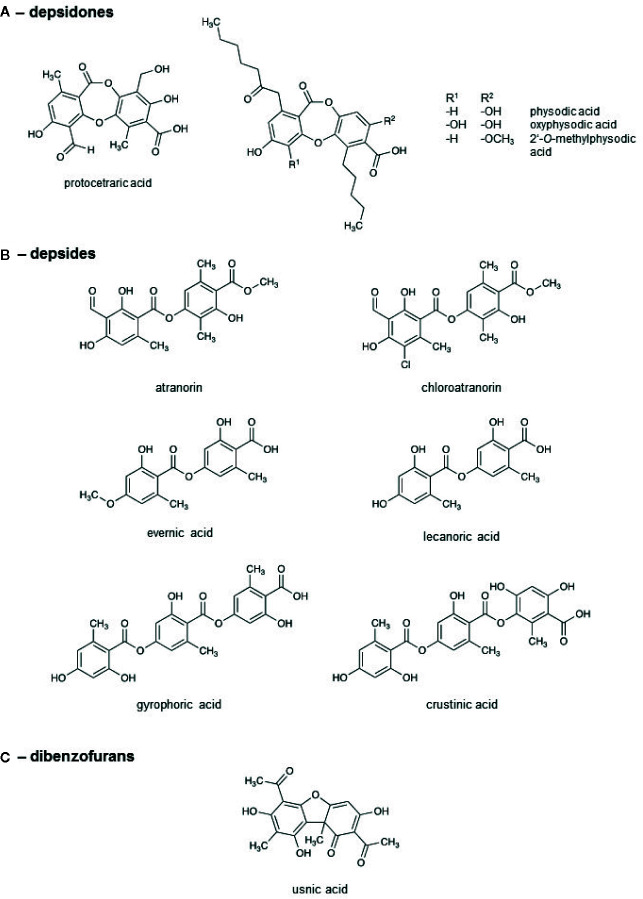
Chemical structures of the lichen compounds.

To get insights into the ingredient profile of each lichen extract, the natural compounds in the lichen extracts were identified by HPLC. The metabolite patterns of the lichen extracts are given in **Table 2** and **Supplemental Figure 5**. The chemical structures of the most relevant molecules are provided in **Figure 4**. *E. prunastri* produced mainly evernic acid. *P*. *furfuracea* synthesized mainly physodic acid, atranorin, 2’-*O*-methylphysodic acid, and oxyphysodic acid. The *U. pustulata* extract contained gyrophoric acid and lecanoric acid, which here is an artificial satellite product (cleavage product of gyrophoric acid produced during extraction and HPLC). The *U. crustulosa* extract contained crustinic acid, gyrophoric acid, and methyl lecanorate, which is a cleavage product of crustinic acid. *F. caperata* extracts contained as main UV-absorbing compounds protocetraric acid and usnic acid, and *P. glauca* extracts contained mainly chloroatranorin and atranorin.

### Correlation Analysis Between Lichen Compounds and Test Systems

**Figure 5:**
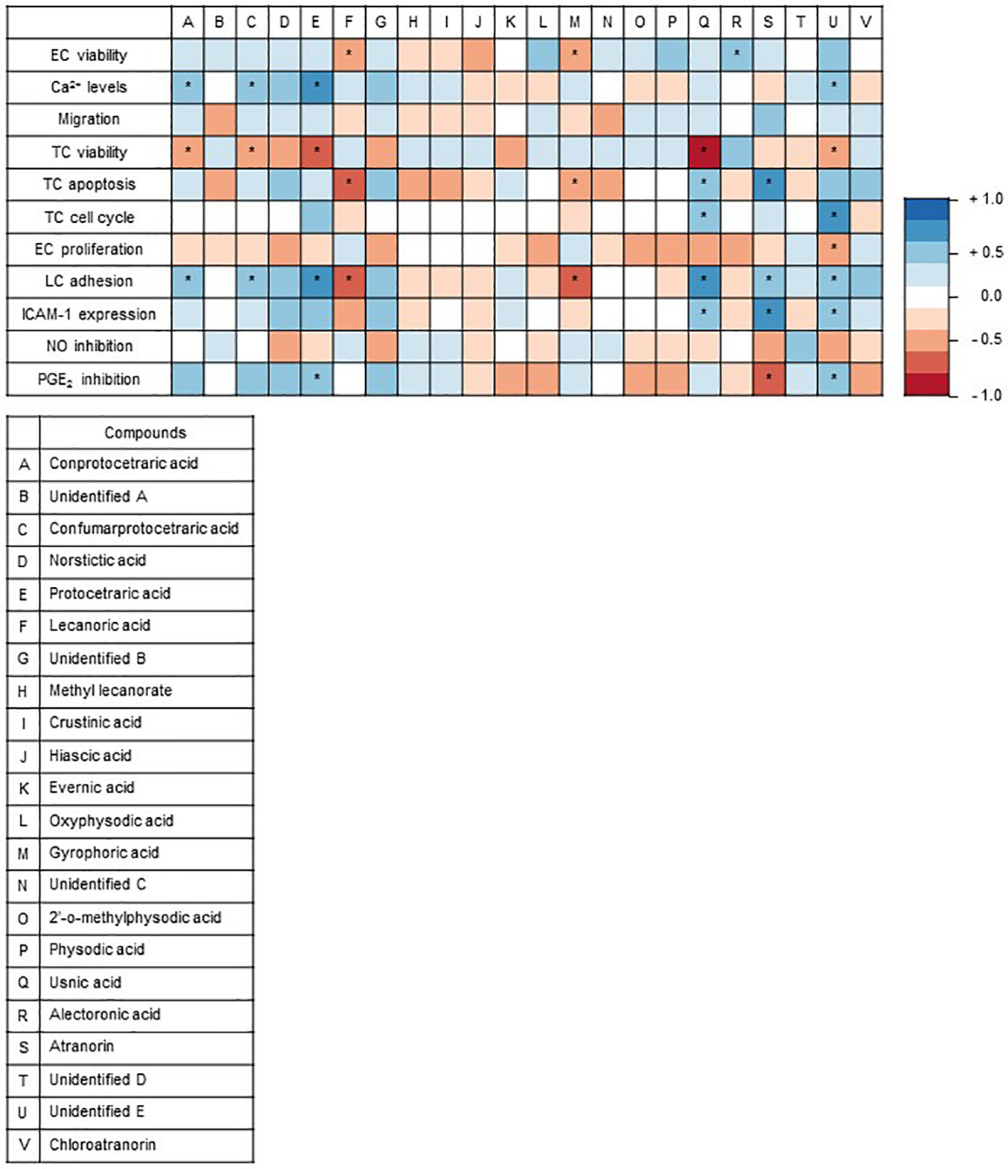
Graphical representation of Spearman’s correlation matrix. The natural compounds detected *via* high performance liquid chromatography (HPLC) in the lichen extracts were correlated with the outcomes of the 11 functional and biochemical test systems. The heat map shows Spearman’s correlation between the outcomes of the test systems and the natural compounds of the lichens identified by HPLC. Each column represents an individual compound and each row defines an individual test system. Positive correlation values are in red, and negative correlation values are in blue. The correlation analysis of lichen substances and assay outcomes was conducted with R (V3.6.1 available online https://www.R-project.org/) (Macdonald et al., 1999) and visualized as correlation matrix (V0.84 available online https://github.com/taiyun/corrplot; ColorBrewer Palettes V1.1-2 available online https://CRAN.R-project.org/package=RColorBrewer). *p ≤ 0.05.

A correlation analysis was used to assign the secondary metabolites identified in the lichen extracts to specific observed effects in the used test systems, since this statistical method is suitable to evaluate the strength of a relationship between two quantitative variables. The correlation analysis was achieved with the results obtained by the higher lichen concentration (30 µg/ml). The colors indicate the type of correlation, where blue indicates a positive correlation and red a negative correlation. The correlation analysis revealed that usnic acid negatively correlates with cell viability and positively with apoptosis (**Figure 5**). Usnic acid was predominantly found in *F. caperata* extract, which evoked an induction of apoptosis and a reduction of cell viability (**Figures 2A, C**). Protocetraric acid is positively correlated to an increased Ca^2+^ level. Protocetraric was detected in *F. caperata*, the lichen extract which induce Ca^2+^ signaling in HEK293 cells (**Figure 1B**). However, further studies needed to confirm these suggestions drawn by the correlation analysis.

## Discussion

**Figure 6:**
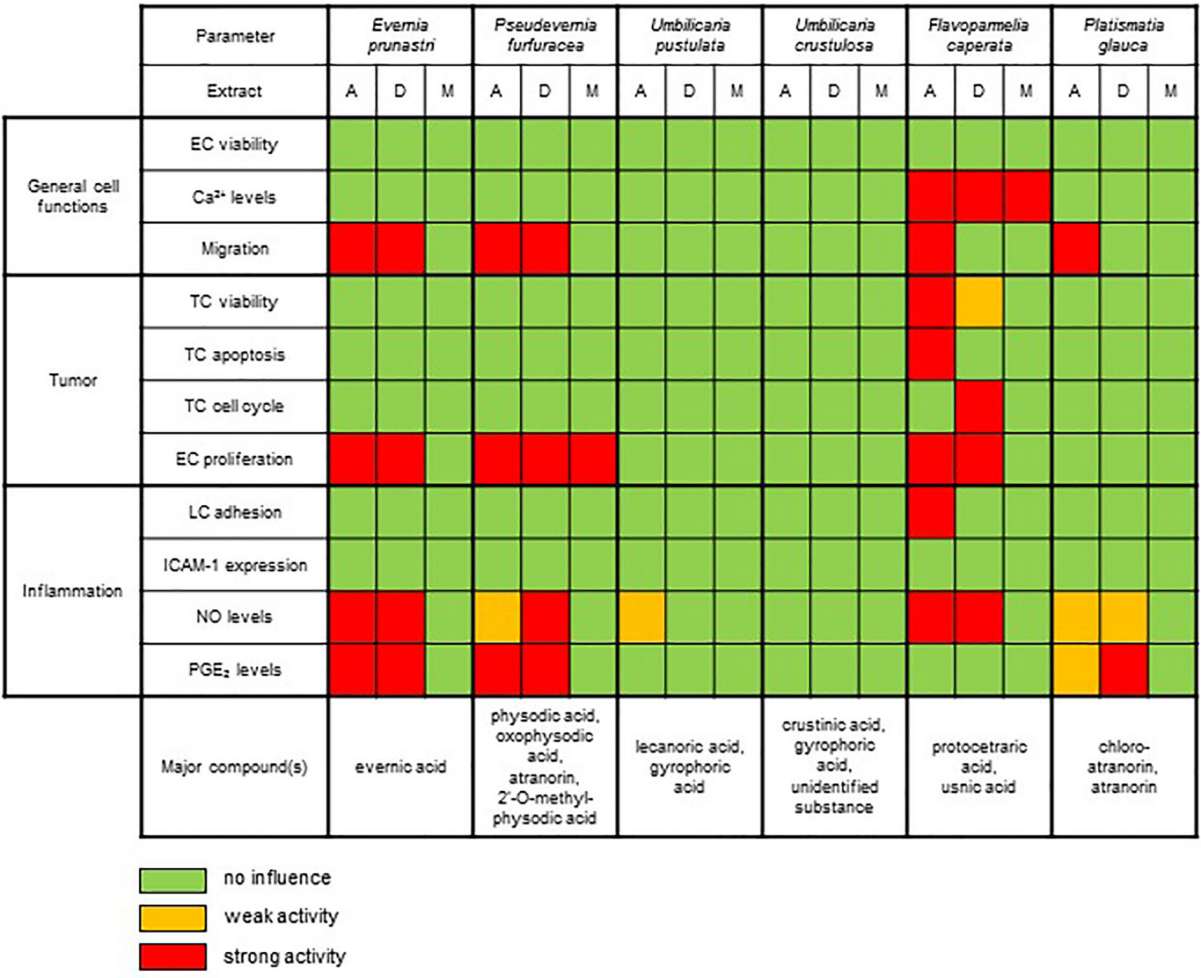
Summary of the effects of the UV-absorbing lichen extracts in the various biochemical and functional test systems.

By applying a broad screening approach consisting of 11 different *in vitro* assays to six different lichen species, we unraveled interesting bioactivity profiles for four of the six species (**Figure 6**). Remarkably, none of the lichen extracts showed any detrimental influence on the viability of healthy (endothelial) cells. However, extracts from *F. caperata* induced cellular Ca^2+^ signaling. To our knowledge, this biological activity is shown here for the first time for a lichen extract. Our results on analyses of *E. prunastri*, *P. furfuracea*, *F. caperata*, and *P. glauca* are in correlation with the previous reports of lichen substances that can reduce the migratory capacity of cells (Burlando et al., 2009; Ebrahim et al., 2016; Zhou et al., 2017; Seklic et al., 2018). Interestingly, extracts from *F. caperata* strongly decreased the survival of tumor cells, which is also in line with studies from others (Mitrovic et al., 2011). The proliferation of ECs—a hallmark of angiogenic processes associated with tumor growth (Hanahan and Weinberg, 2000)—was significantly reduced by extracts from *E. prunastri*, *P. furfuracea*, and *F. caperata*. In the context of inflammation, the lichen extracts had no inhibitory activity on inflammatory responses in endothelial cells. However, the pro-inflammatory activation of leukocytes was substantially reduced by extracts from *F. caperata*, *E. prunastri*, *P. furfuracea*, and *P. glauca*. The anti-inflammatory activity might have resulted from the abundant secondary metabolites found in these species. For example, the anti-inflammatory effects of a substance, usnic acid, which is present in *F. caperata* (major) and *E. prunastri* (minor) (Vijayakumar et al., 2000), and also from a lichen extract containing atranorin and chloroatranorin (Bugni et al., 2009), substances present in *P. furfuracea* and *P. glauca*, were previously reported by others.

To investigate further, HPLC is preferred as a modern analytical method to perform fast and high-resolution identification of lichen compounds. The spectra were obtained by a diode array detector that measures the absorbance of light of fixed wavelength in the UV region, which can be taken as the limitation of the applied method (Ratanathanawongs and Crouch, 1987) in this study. Besides, the standardization of the raw lichen material is usually a challenging step due to the alterations depending on environmental and seasonal stimuli (Bertoli et al., 2010).

In this study, we focused on detecting the presence of known UV-absorbing secondary metabolites and their relative proportions, rather than finding their precise concentrations. The UV-absorbing natural compounds identified by HPLC (**Table 2**) can be assigned to three substance classes (**Figure 4**), the depsidones, depsides, and dibenzofurans. The lichens can be classified into three groups: lichens with predominantly depsidones and depsides (*P. furfuracea*), with predominantly depsides (*E. prunastri*, *U. pustulata*, *U. crustulosa*, and *P. glauca*), with a depsidone and a dibenzofuran (*F. carperata*) as the most abundant compounds. However, we found no common activity profiles for these three groups, suggesting that individual compounds rather than substance classes are responsible for the observed effects.

As one of the goals, data obtained from the *in vitro* screening approaches were correlated with known secondary metabolites. The correlation analysis (**Figure 5**) assigned specific compounds to the tested cellular functions. The analysis data suggested that protocetraric acid could be the reason of the increase in Ca^2+^ level and reduced tumor cell viability. In the literature not much is known about protocetraric acid beside its effect on cell proliferation. Previously, Brando *et al*. indicated that protocetraric acid strongly inhibits the growth of the melanoma cell line UACC-62 (IC_50_: 0.5 µg/ml) (Brandao et al., 2013), moderately the growth of the melanoma cell line B16-F10 (IC_50_: 24 µg/ml) (Brandao et al., 2013), the melanoma cell line FemX (IC_50_: ≈ 50 µM) (Manojlovic et al., 2012), and the colon carcinoma cell line LS174 (IC_50_: ≈ 50 µM) (Manojlovic et al., 2012), while it has no effect on NIH/3T3 fibroblasts (Brandao et al., 2013). This is in line with our findings, since *F. caperata* (lichen with protocetraric acid) has no effect on HMECs but showed a reduction of viability in the colon cancer cell line HCT-116 suggesting that protocetraric is a potential new drug candidate for cancer due its selectivity for cancer cells. Furthermore, the correlation analysis also found that usnic acid might be responsible from the reduced tumor cell viability, apoptosis, and cell cycle inhibition. In contrast to protocetraric acid, usnic acid as lead compound of *F. caperata*, has various well-known activities (Cocchietto et al., 2002; Ingolfsdottir, 2002; Guzow-Krzemińska et al., 2019). In line with our findings, it was already shown that usnic acid induce a G1-Block in the gastric cancer cell line BGC823, in A549 lung carcinoma cells (Singh et al., 2013), in the breast cancer cell line MCF-7, and in the prostate adenocarcinoma cell line LNCap (Eryilmaz et al., 2018; Geng et al., 2018). However, in the gastric cancer cell line SGC7901, a G2-Block was induced (Geng et al., 2018) and Backorová showed that usnic acid (100 µM) induces an S-Block in HCT-116 cells. These data indicate that usnic acid does not consistently induce cell cycle blocks in all cells and possibly acts through different signaling pathways in various cell types. Accordingly, usnic acid could well be responsible for the observed G1-Block induced by the *F. caperata* extract in HCT-116 cells. Besides the cell cycle block, the extract of *F. caperata* also induced apoptosis *via* caspase 3 activation that is possible also due to usnic acid since Bačkorová *et al*. showed that usnic acid induced in the colon carcinoma cell line HT29 apoptosis *via* caspase 3 activation (Backorova et al., 2012). Our data suggested an anti-carcinogenic potential of usnic acid as it was already shown by Mitrovic *et al*. (Mitrovic et al., 2011). Usnic acid was recently described to possess anti-angiogenic properties (Song et al., 2012; Koparal, 2015; Song et al., 2018), which is in line with the observed anti-proliferative actions in the current study. Whether the major compounds of the extract from *E. prunastri*, *P. furfuracea*, and *F. caperata* that inhibit EC proliferation—namely protocetraric acid, physodic acid (or its derivatives), and evernic acid—are responsible for the effects on EC proliferation is not known and warrants further studies.

Moreover, the correlation analysis suggests that atranorin and physodic acid and its derivatives may inhibit the LPS-induced PGE_2_ production, whereas for the inhibition of NO synthesis no significant correlation was found. Only few studies addressed the effect of lichens on the NO and PGE_2_ synthesis. Physodic acid inhibited PGE_2_ synthesis in a cell free assay (Bauer et al., 2012), whereas atranorin has not yet been investigated for effects on PGE_2_ synthesis. Usnic acid has already been shown to inhibit NO synthesis *in vivo* (Odabasoglu et al., 2006) and *in vitro* (Jin et al., 2008). Therefore, our correlation analysis results point out atranorin and physodic acid as promising secondary metabolites with potential anti-inflammatory properties.

To conclude, our biochemical and functional assay arrays revealed that some of the lichen extracts have significant pharmacological activity in the context of cancer and/or inflammation. Furthermore, our correlation analysis suggests some lead compounds such as protocetraric acid, usnic acid, and atranorin as potent natural components of lichen extracts, which should be further investigated regarding their suitability as new drug candidates.

## Data Availability Statement

The datasets generated for this study are available on request to the corresponding author.

## Author Contributions

IS, RF, SS, and GG designed the study and/or wrote the manuscript. RI, TU, MH, AC, and GS achieved the experiments and/or analyzed the data. MP, RF, IS, RI, and SS revised the manuscript. All authors contributed to the article and approved the submitted version.

## Funding

This work was supported by the Landesoffensive zur Entwicklung wissenschaftlich-ökonomischer Exzellenz (LOEWE), Center “Translationale Medizin und Pharmakologie” (TMP) and the LOEWE Center “Novel Drug Targets against Poverty-Related and Neglected Tropical Infectious Diseases” (DRUID) and the LOEWE Center “Translational Biodiversity Genomics” (TBG).

## Conflict of Interest

The authors declare that the research was conducted in the absence of any commercial or financial relationships that could be construed as a potential conflict of interest.

## Abbreviations

A, acetone extract; ANOVA, analysis of variance; D, dichloromethane extract; EC, endothelial cell; FBS, fetal bovine serum; HUVEC, human umbilical vein endothelial cells; ICAM-1, intercellular adhesion molecule 1; M, 70% methanol extract; NO, nitric oxide; PBS, phosphate-buffered saline; PGE_2_, prostaglandin E_2_.

## References

[B1] Ades E. W. Candal F. J. Swerlick R. A. George V. G. Summers S. Bosse D. C. (1992). HMEC-1: establishment of an immortalized human microvascular endothelial cell line. J. Invest. Dermatol. 99 (6), 683–690. 10.1111/1523-1747.ep12613748 1361507

[B2] Asplund J. Bokhorst S. Kardol P. Wardle D. A. (2015). Removal of secondary compounds increases invertebrate abundance in lichens. Fungal Ecol. 18, 18–25. 10.1016/j.funeco.2015.07.009

[B3] Backorova M. Jendzelovsky R. Kello M. Backor M. Mikes J. Fedorocko P. (2012). Lichen secondary metabolites are responsible for induction of apoptosis in HT-29 and A2780 human cancer cell lines. Toxicol. Vitro 26 (3), 462–468. 10.1016/j.tiv.2012.01.017 22285236

[B4] Bauer J. Waltenberger B. Noha S. M. Schuster D. Rollinger J. M. Boustie J. (2012). Discovery of depsides and depsidones from lichen as potent inhibitors of microsomal prostaglandin E2 synthase-1 using pharmacophore models. ChemMedChem 7 (12), 2077–2081. 10.1002/cmdc.201200345 23109349 PMC3524419

[B5] Benatti M. N. Gernert M. Schmitt I. (2013). Parmotrema hydrium, a new species of Parmeliaceae in southeastern Brazil. Acta Botanica Brasilica 27 (4), 810–814. 10.1590/S0102-33062013000400021

[B6] Bertoli A. Ruffoni B. Pistelli L. Pistelli L. (2010). Analytical methods for the extraction and identification of secondary metabolite production in ‘in vitro’ plant cell cultures. Adv. Exp. Med. Biol. 698, 250–266. 10.1007/978-1-4419-7347-4_19 21520717

[B7] Brandao L. F. Alcantara G. B. Matos Mde F. Bogo D. Freitas Ddos S. Oyama N. M. (2013). Cytotoxic evaluation of phenolic compounds from lichens against melanoma cells. Chem. Pharm. Bull. (Tokyo) 61 (2), 176–183. 10.1248/cpb.c12-00739 23207680

[B8] Bugni T. S. Andjelic C. D. Pole A. R. Rai P. Ireland C. M. Barrows L. R. (2009). Biologically active components of a Papua New Guinea analgesic and anti-inflammatory lichen preparation. Fitoterapia 80 (5), 270–273. 10.1016/j.fitote.2009.03.003 19289158 PMC2793093

[B9] Burlando B. Ranzato E. Volante A. Appendino G. Pollastro F. Verotta L. (2009). Antiproliferative effects on tumour cells and promotion of keratinocyte wound healing by different lichen compounds. Planta Med. 75 (6), 607–613. 10.1055/s-0029-1185329 19199230

[B10] Cocchietto M. Skert N. Nimis P. L. Sava G. (2002). A review on usnic acid, an interesting natural compound. Naturwissenschaften 89 (4), 137–146. 10.1007/s00114-002-0305-3 12061397

[B11] Crawford S. D. (2019). “Lichens Used in Traditional Medicine,” in Lichen Secondary Metabolites. Ed. Ranković B. (Cham Germany: Springer).

[B12] Cui C. Merritt R. Fu L. Pan Z. (2017). Targeting calcium signaling in cancer therapy. Acta Pharm. Sin. B 7 (1), 3–17. 10.1016/j.apsb.2016.11.001 28119804 PMC5237760

[B13] Ebrahim H. Y. Elsayed H. E. Mohyeldin M. M. Akl M. R. Bhattacharjee J. Egbert S. (2016). Norstictic Acid Inhibits Breast Cancer Cell Proliferation, Migration, Invasion, and In Vivo Invasive Growth Through Targeting C-Met. Phytother. Res. 30 (4), 557–566. 10.1002/ptr.5551 26744260 PMC5045260

[B14] Elix J. A. Stocker-Wörgötter E. (2008). “Biochemistry and secondary metabolites,” in III: Lichen Biology, 2nd ed. Ed. Nash T. H. (Cambridge: Cambridge University Press), 104–133.

[B15] Eryilmaz I. E. Guney Eskiler G. Egeli U. Yurdacan B. Cecener G. Tunca B. (2018). In vitro cytotoxic and antiproliferative effects of usnic acid on hormone-dependent breast and prostate cancer cells. J. Biochem. Mol. Toxicol. 32 (10), e22208. 10.1002/jbt.22208 30101414

[B16] Feige G. B. Lumbsch H. T. Huneck S. Elix J. A. (1993). Identification of lichen substances by a standardized high-performance liquid chromatographic method. J. Chromatogr. A 646 (2), 417–427. 10.1016/0021-9673(93)83356-W

[B17] Gauslaa Y. (2005). Lichen palatability depends on investments in herbivore defence. Oecologia 143 (1), 94–105. 10.1007/s00442-004-1768-z 15619096

[B18] Geng X. Zhang X. Zhou B. Zhang C. Tu J. Chen X. (2018). Usnic Acid Induces Cycle Arrest, Apoptosis, and Autophagy in Gastric Cancer Cells In Vitro and In Vivo. Med. Sci. Monit. 24, 556–566. 10.12659/msm.908568 29374767 PMC5798279

[B19] Guzow-Krzemińska B. Guzow K. Herman-Antosiewicz A. (2019). Usnic Acid Derivatives as Cytotoxic Agents Against Cancer Cells and the Mechanisms of Their Activity. Curr. Pharmacol. Rep. 5, 429–439. 10.1007/s40495-019-00202-8

[B20] Hanahan D. Weinberg A. (2000). The Hallmarks of Cancer. Cell 100, 57–70. 10.1016/S0092-8674(00)81683-9 10647931

[B21] Huneck S. Yoshimura I. (1996). Identification of Lichen Substances. (Berlin, Heidelberg: Springer-Verlag) 493.

[B22] Huneck S. (2003). “Die wasserabweisende Eigenschaft von Flechtenstoffen,” in Lichenological Contributions in Honour of G.B. Feige. Ed. Jansen M. (J. Cramer Berlin, Stuttgart: In der Gebrüder Bornträger Verlagsbuchhandlung), 9–12.

[B23] Ingolfsdottir K. (2002). Usnic acid. Phytochemistry 61 (7), 729–736. 10.1016/s0031-9422(02)00383-7 12453567

[B24] Jin J. Q. Li C. Q. He L. C. (2008). Down-regulatory effect of usnic acid on nuclear factor-kappaB-dependent tumor necrosis factor-alpha and inducible nitric oxide synthase expression in lipopolysaccharide-stimulated macrophages RAW 264.7. Phytother. Res. 22 (12), 1605–1609. 10.1002/ptr.2531 19003951

[B25] Koparal A. T. (2015). Anti-angiogenic and antiproliferative properties of the lichen substances (-)-usnic acid and vulpinic acid. Z Naturforsch. C J. Biosci. 70 (5-6), 159–164. 10.1515/znc-2014-4178 26136299

[B26] Macdonald J. M. LeBlanc D. A. Haas A. L. London R. E. (1999). An NMR analysis of the reaction of ubiquitin with [acetyl-1-13C]aspirin. Biochem. Pharmacol. 57 (11), 1233–1244. 10.1016/S0006-2952(99)00039-8 10230767

[B27] Manojlovic N. Rankovic B. Kosanic M. Vasiljevic P. Stanojkovic T. (2012). Chemical composition of three Parmelia lichens and antioxidant, antimicrobial and cytotoxic activities of some their major metabolites. Phytomedicine 19 (13), 1166–1172. 10.1016/j.phymed.2012.07.012 22921748

[B28] Mitrovic T. Stamenkovic S. Cvetkovic V. Tosic S. Stankovic M. Radojevic I. (2011). Antioxidant, antimicrobial and antiproliferative activities of five lichen species. Int. J. Mol. Sci. 12 (8), 5428–5448. 10.3390/ijms12085428 21954369 PMC3179176

[B29] Molnar K. Farkas E. (2010). Current results on biological activities of lichen secondary metabolites: a review. Z Naturforsch. C 65 (3-4), 157–173. 10.1515/znc-2010-3-401 20469633

[B30] Odabasoglu F. Cakir A. Suleyman H. Aslan A. Bayir Y. Halici M. (2006). Gastroprotective and antioxidant effects of usnic acid on indomethacin-induced gastric ulcer in rats. J. Ethnopharmacol. 103 (1), 59–65. 10.1016/j.jep.2005.06.043 16169175

[B31] Porter A. G. Janicke R. U. (1999). Emerging roles of caspase-3 in apoptosis. Cell Death Differ. 6 (2), 99–104. 10.1038/sj.cdd.4400476 10200555

[B32] Rankovic B. Kosanic M. (2014). “Lichens as a Potential Source of Bioactive Secondary Metabolites,” in *Lichen Secondary Metabolites Bioactive Properties and Pharmaceutical Potential* . Ed. Ranković B. (Switzerland: Springer International Publishing), 1–26. 10.1007/978-3-319-13374-4_1

[B33] Ratanathanawongs S. K. Crouch S. R. (1987). Development of a selective post-column detector for phenols separated by high-performance liquid chromatography. Analytica Chim. Acta 192, 277–287. 10.1016/S0003-2670(00)85712-8

[B34] Schöller H. (1997). Flechten. Kleine Senckenbergreihe Nr 27, 1–247.

[B35] Seklic D. S. Obradovic A. D. Stankovic M. S. Zivanovic M. N. Mitrovic T. L. Stamenkovic S. M. (2018). Proapoptotic and Antimigratory Effects of Pseudevernia furfuracea and Platismatia glauca on Colon Cancer Cell Lines. Food Technol. Biotechnol. 56 (3), 421–430. 10.17113/ftb.56.03.18.5727 30510485 PMC6233007

[B36] Serina E. Arroyo R. Manrique E. Sancho L. G. Serina E. (1996). Lichen Substances and Their Intraspecifie Variability within Eleven Umbilicaria Species in Spain. Bryologist 99 (3), 335–342. 10.2307/3244307

[B37] Singh N. Nambiar D. Kale R. K. Singh R. P. (2013). Usnic acid inhibits growth and induces cell cycle arrest and apoptosis in human lung carcinoma A549 cells. Nutr. Cancer 65 (Suppl 1), 36–43. 10.1080/01635581.2013.785007 23682781

[B38] Solhaug K. A. Gauslaa Y. Nybakken L. Bilger W. (2003). UV-induction of sun-screening pigments in lichens. New Phytol. 158 (1), 91–100. 10.1046/j.1469-8137.2003.00708.x

[B39] Song Y. Dai F. Zhai D. Dong Y. Zhang J. Lu B. (2012). Usnic acid inhibits breast tumor angiogenesis and growth by suppressing VEGFR2-mediated AKT and ERK1/2 signaling pathways. Angiogenesis 15 (3), 421–432. 10.1007/s10456-012-9270-4 22669534

[B40] Song Y. Yu Z. Song B. Guo S. Lei L. Ma X. (2018). Usnic acid inhibits hypertrophic scarring in a rabbit ear model by suppressing scar tissue angiogenesis. BioMed. Pharmacother. 108, 524–530. 10.1016/j.biopha.2018.06.176 30243085

[B41] Trepat X. Chen Z. Jacobson K. (2012). Cell migration. Compr. Physiol. 2 (4), 2369–2392. 10.1002/cphy.c110012 23720251 PMC4457291

[B42] Tsuchiya S. Yamabe M. Yamaguchi Y. Kobayashi Y. Konno T. Tada K. (1980). Establishment and characterization of a human acute monocytic leukemia cell line (THP-1). Int. J. Cancer 26 (2), 171–176. 10.1002/ijc.2910260208 6970727

[B43] Vijayakumar C. S. Viswanathan S. Reddy M. K. Parvathavarthini S. Kundu A. B. Sukumar E. (2000). Anti-inflammatory activity of (+)-usnic acid. Fitoterapia 71 (5), 564–566. 10.1016/s0367-326x(00)00209-4 11449509

[B44] Wirtz D. Konstantopoulos K. Searson P. C. (2011). The physics of cancer: the role of physical interactions and mechanical forces in metastasis. Nat. Rev. Cancer 11 (7), 512–522. 10.1038/nrc3080 21701513 PMC3262453

[B45] Zhou R. Yang Y. Park S. Y. Nguyen T. T. Seo Y. W. Lee K. H. (2017). The lichen secondary metabolite atranorin suppresses lung cancer cell motility and tumorigenesis. Sci. Rep. 7 (1), 8136. 10.1038/s41598-017-08225-1 28811522 PMC5557893

