# Supplementary material for: Unraveling the Pharmacological Potential of Lichen Extracts in the Context of Cancer and Inflammation With a Broad Screening Approach
Source: Front Pharmacol. 2020 Sep 4;11:1322. doi: 10.3389/fphar.2020.01322 (PMC7509413; doi:10.3389/fphar.2020.01322)
Supplement: Supplementary file 1 [file Table_1.docx]

| **Species name** | **Specimen number** | **HPLC number (Acetone extract)** | **Voucher information** | **Herbarium accession number** |
| --- | --- | --- | --- | --- |
| *Evernia prunastri* | 3108 | H19-001_08A | France, Corsica, near village of Vico, by road side of road D70 from Sagone to Vico, 274m, 42.1523 lat, 8.7587 long, 02.05.2018, leg. Imke Schmitt | FR-0265616 |
| *Evernia prunastri* | 3121 | H19-001_08B | France, Corsica, near village of Vico, by road side of road D70 from Sagone to Vico, 274m, 42.1523 lat, 8.7587 long, 02.05.2018, leg. Imke Schmitt | FR-0265617 |
| *Pseudevernia furfuracea* | 3325 | H19-001_08C | France, Corsica, Foret D'Aitone, east of village of Evisa, by road side of road D84 through Foret Territoriale d'Aitone, 1109m, 42.26988 lat, 8.84304 long, 02.05.2018, leg. Imke Schmitt | FR-0265618 |
| *Pseudevernia furfuracea* | 3332 | H19-001_08D | France, Corsica, Foret D'Aitone, east of village of Evisa, by road side of road road D84 through Foret Territoriale d'Aitone, 1109m, 42.26988 lat, 8.84304 long, 02.05.2018, leg. Imke Schmitt | FR-0265619 |
| *Umbilicaria crustulosa* | 4128 | H19-001_08E | France, Corsica, near village of Carbini, near road D59, rocky outcrops NE of village, 553m, 41.68175 lat, 9.14851 long, 07.05.2018, leg. Imke Schmitt | FR-0265620 |
| *Umbilicaria crustulosa* | 4129 | H19-001_08F | France, Corsica, near village of Carbini, near road D59, rocky outcrops NE of village, 553m, 41.68175 lat, 9.14851 long, 07.05.2018, leg. Imke Schmitt | FR-0265621 |
| *Flavoparmelia caperata* | 3636 | H19-001_08G | France, Corsica, near village of Tavera, by road side of road T20, south of branch-off to Tavera, 306m, 42.06065 lat, 8.96931 long, 04.05.2018, leg. Imke Schmitt | FR-0265622 |
| *Flavoparmelia caperata* | 3656 | H19-001_08H | France, Corsica, near village of Tavera, by road side of road T20, south of branch-off to Tavera, 306m, 42.06065 lat, 8.96931 long, 04.05.2018, leg. Imke Schmitt | FR-0265623 |
| *Platismatia glauca* | 4232 | H19-001_09A | France, Corsica, Col de Bavella, above uppermost parking lot, on pine trees, 1230m, 41.79522 lat, 9.22488 long, 09.05.2018, leg. Imke Schmitt | FR-0265624 |
| *Platismatia glauca* | 4237 | H19-001_09B | France, Corsica, Col de Bavella, above uppermost parking lot, on pine trees, 1230m, 41.79522 lat, 9.22488 long, 09.05.2018, leg. Imke Schmitt | FR-0265625 |
| *Umbilicaria pustulata* | 4145 | H19-002_01G | France, Corsica, near village of Carbini, near road D59, rocky outcrops NE of village, 553m, 41.68175 lat, 9.14851 long, 07.05.2018, leg. Imke Schmitt | FR-0265626 |
| *Umbilicaria pustulata* | 4183 | H19-002_02B | France, Corsica, near village of Carbini, near road D59, rocky outcrops NE of village, 553m, 41.68175 lat, 9.14851 long, 07.05.2018, leg. Imke Schmitt | FR-0265627 |

**Supplementary Figure 1**: Specimen information for all samples, including herbarium accession number.
