# Supplementary material for: Unraveling the Pharmacological Potential of Lichen Extracts in the Context of Cancer and Inflammation With a Broad Screening Approach
Source: Front Pharmacol. 2020 Sep 4;11:1322. doi: 10.3389/fphar.2020.01322 (PMC7509413; doi:10.3389/fphar.2020.01322)
Supplement: Supplementary file 2 [file DataSheet_1.pdf]

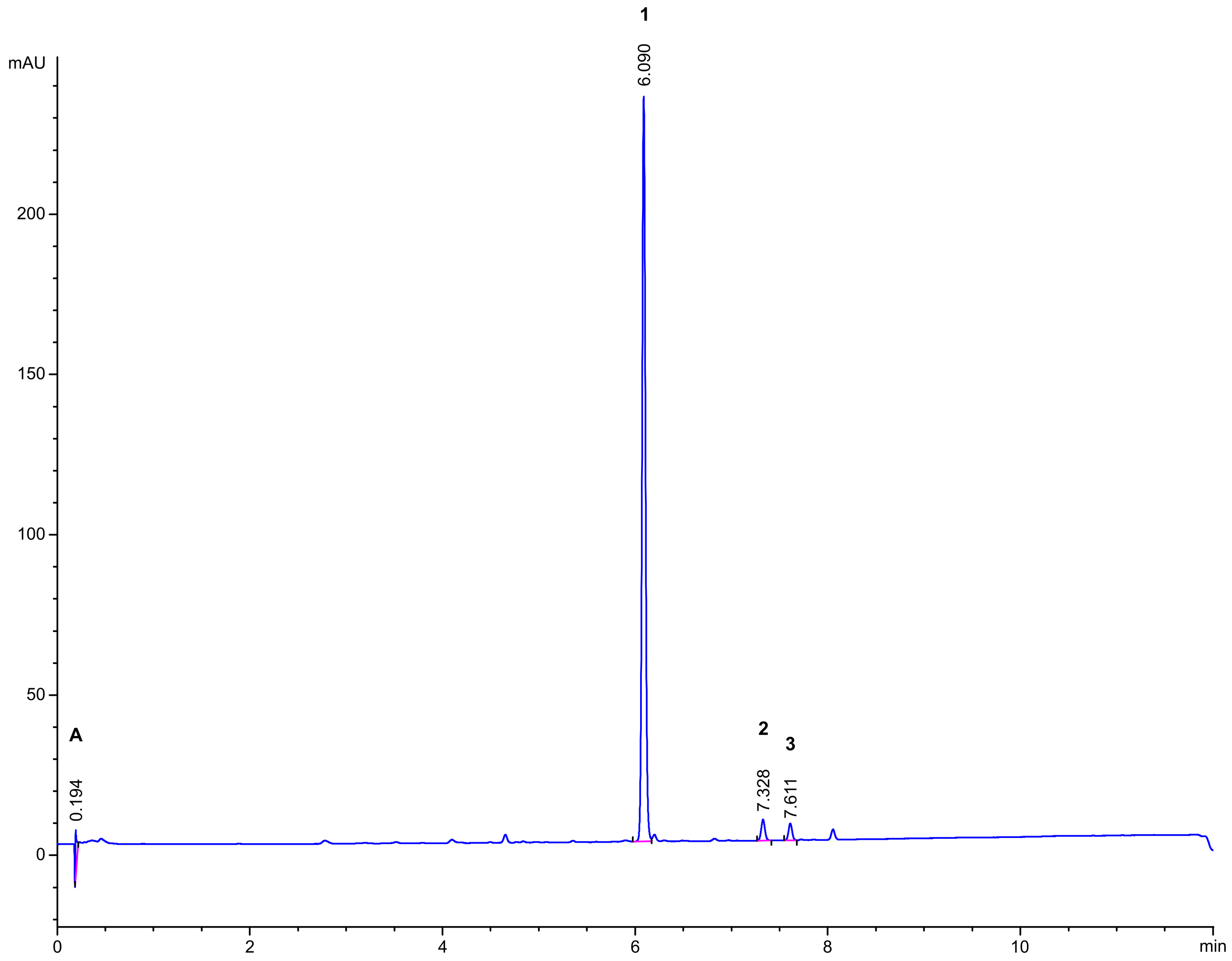


**Figure S1.** Chromatographic analysis (HPLC) of an acetone extract of *Evernia prunastri* (sample number 3108) at 254 nm. A = Acetone; 1 = Evernic acid; 2 = Usnic acid; 3 = Atranorin; mAU = milli-absorbance unit.

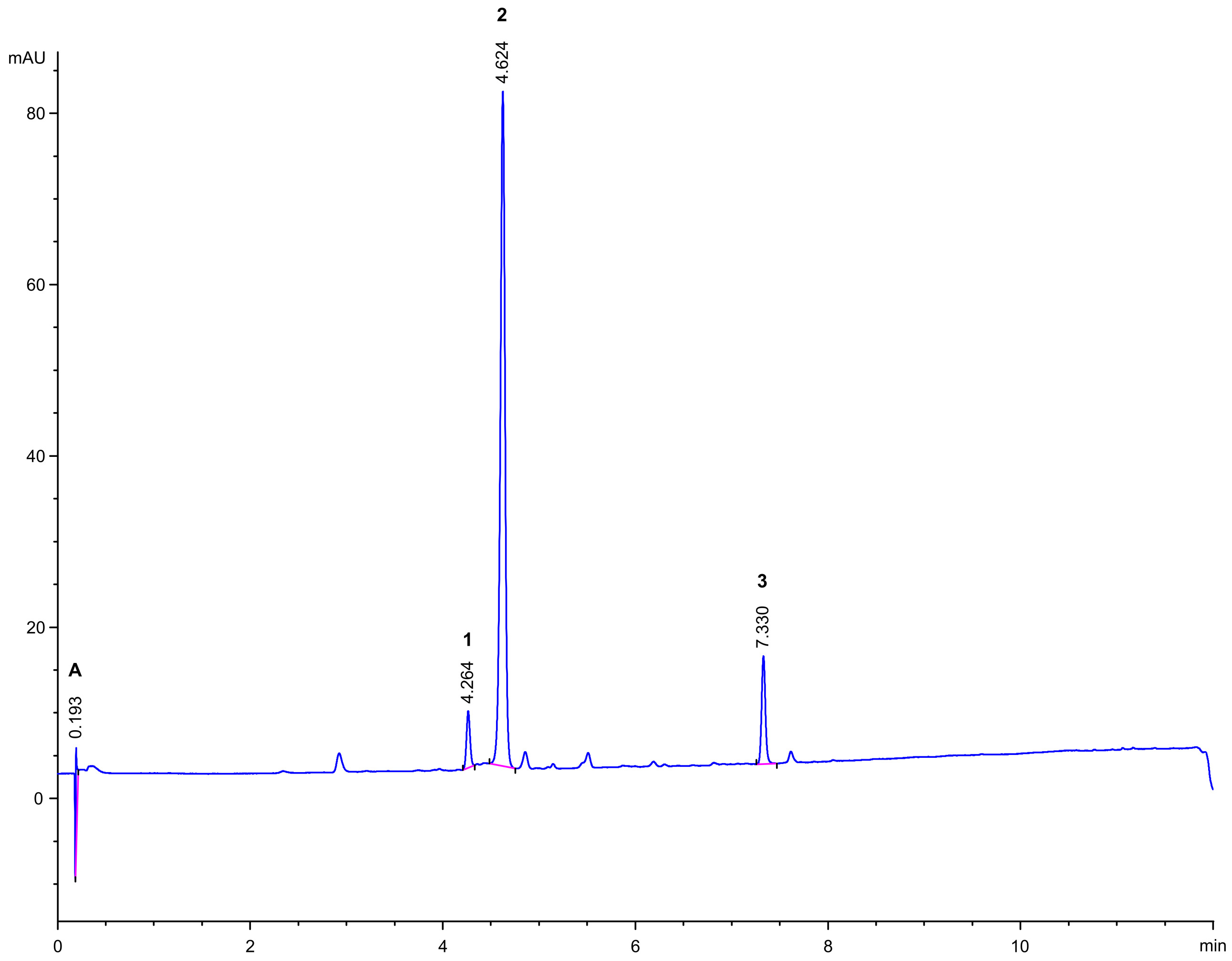


**Figure S2.** Chromatographic analysis (HPLC) of an acetone extract of *Flavoparmelia caperata* (sample number 3636) at 254 nm. A = Acetone; 1 = Confumarprotocetraric acid; 2 = Protocetraric acid; 3 = Usnic acid; mAU = milli-absorbance unit.

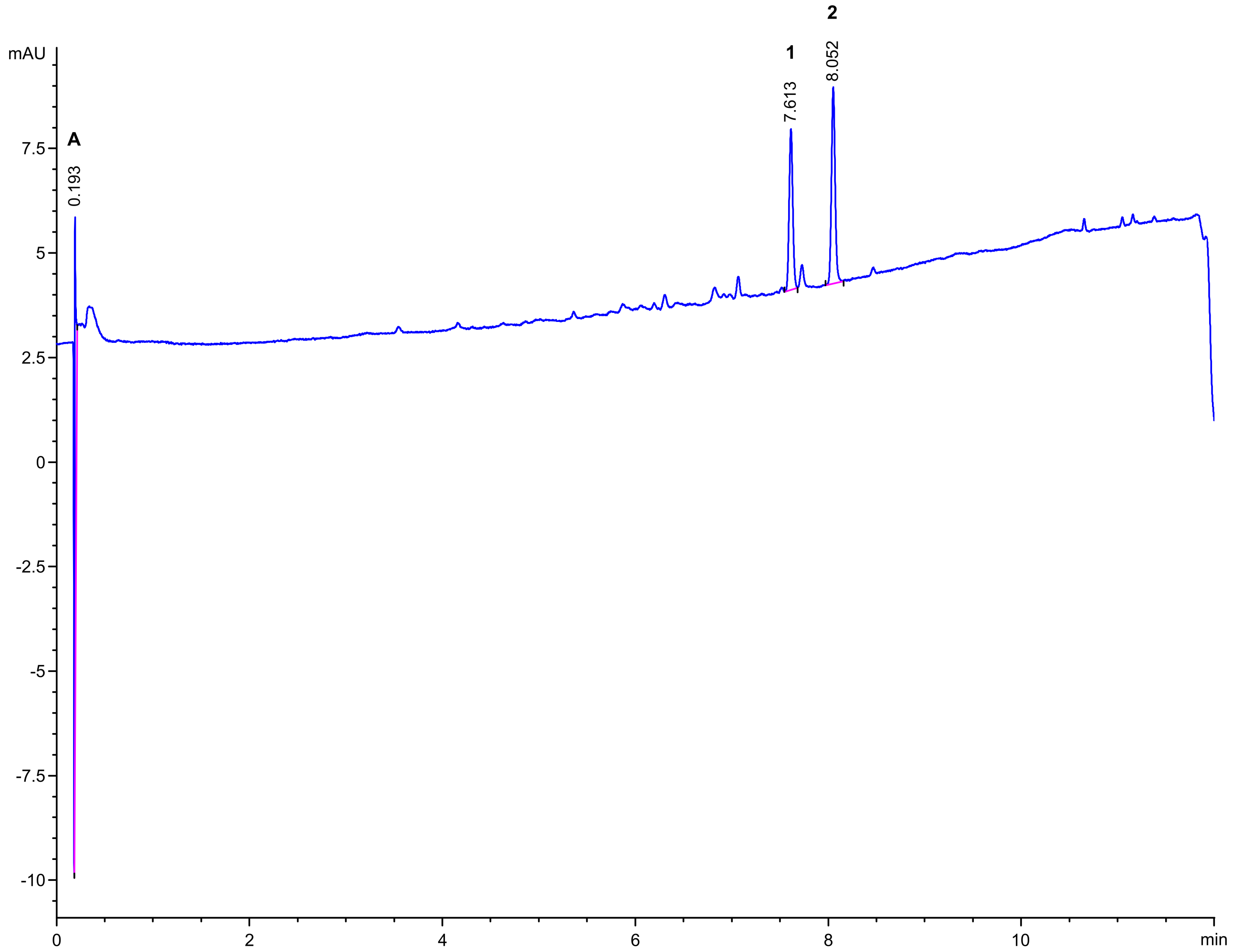


**Figure S3.** Chromatographic analysis (HPLC) of an acetone extract of *Platismatica glauca* (sample number 4232) at 254 nm. A = Acetone; 1 = Atranorin; 2 = Chloroatranorin; mAU = milli-absorbance unit.

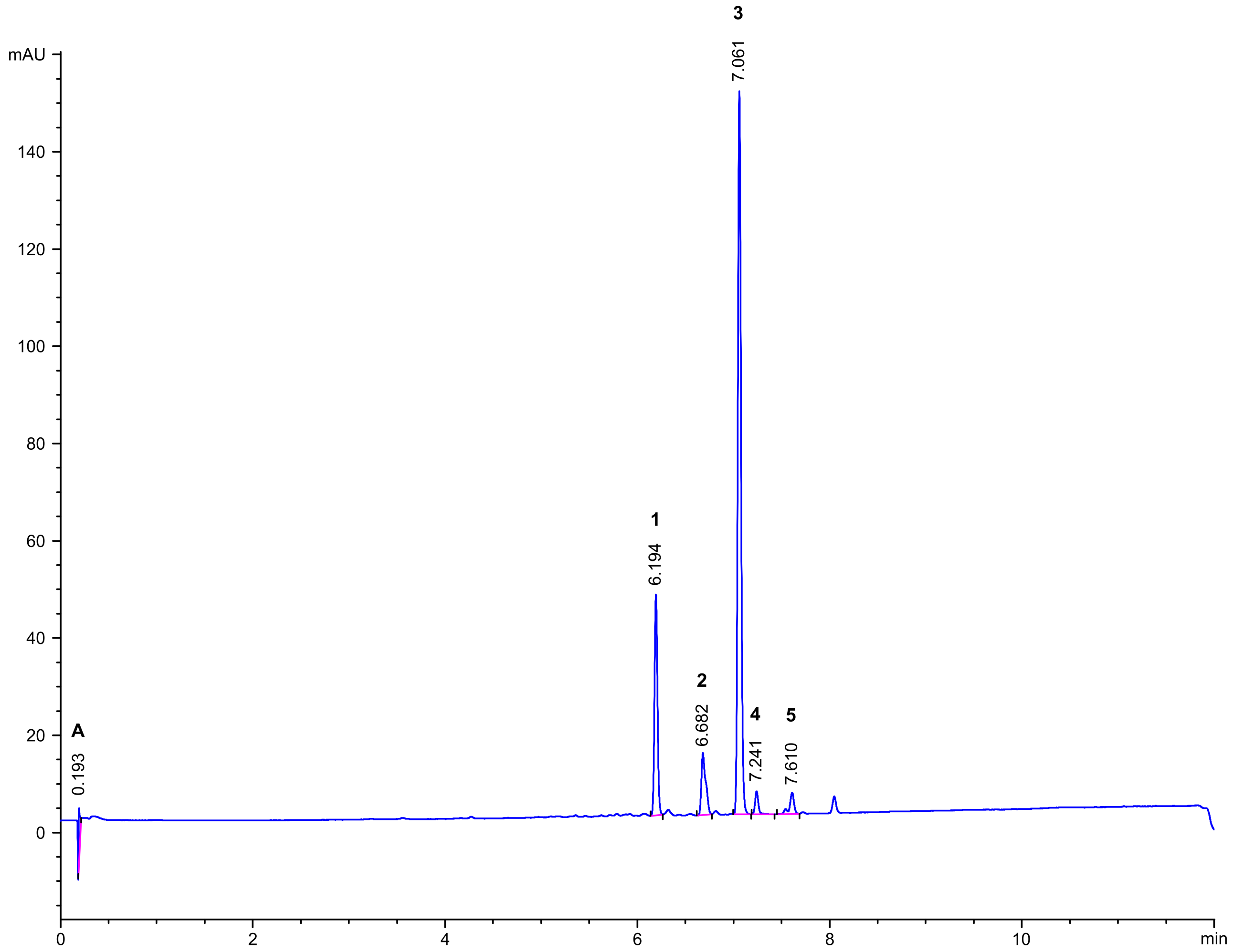


**Figure S4.** Chromatographic analysis (HPLC) of an acetone extract of *Pseudevernia furfuracea* (sample number 3325) at 254 nm. A = Acetone; 1 = Oxyphysodic acid; 2 = 2'-O-Methylphysodic acid; 3 = Physodic acid; 4 = Alectoronic acid; 5 = Atranorin; mAU = milli-absorbance unit.

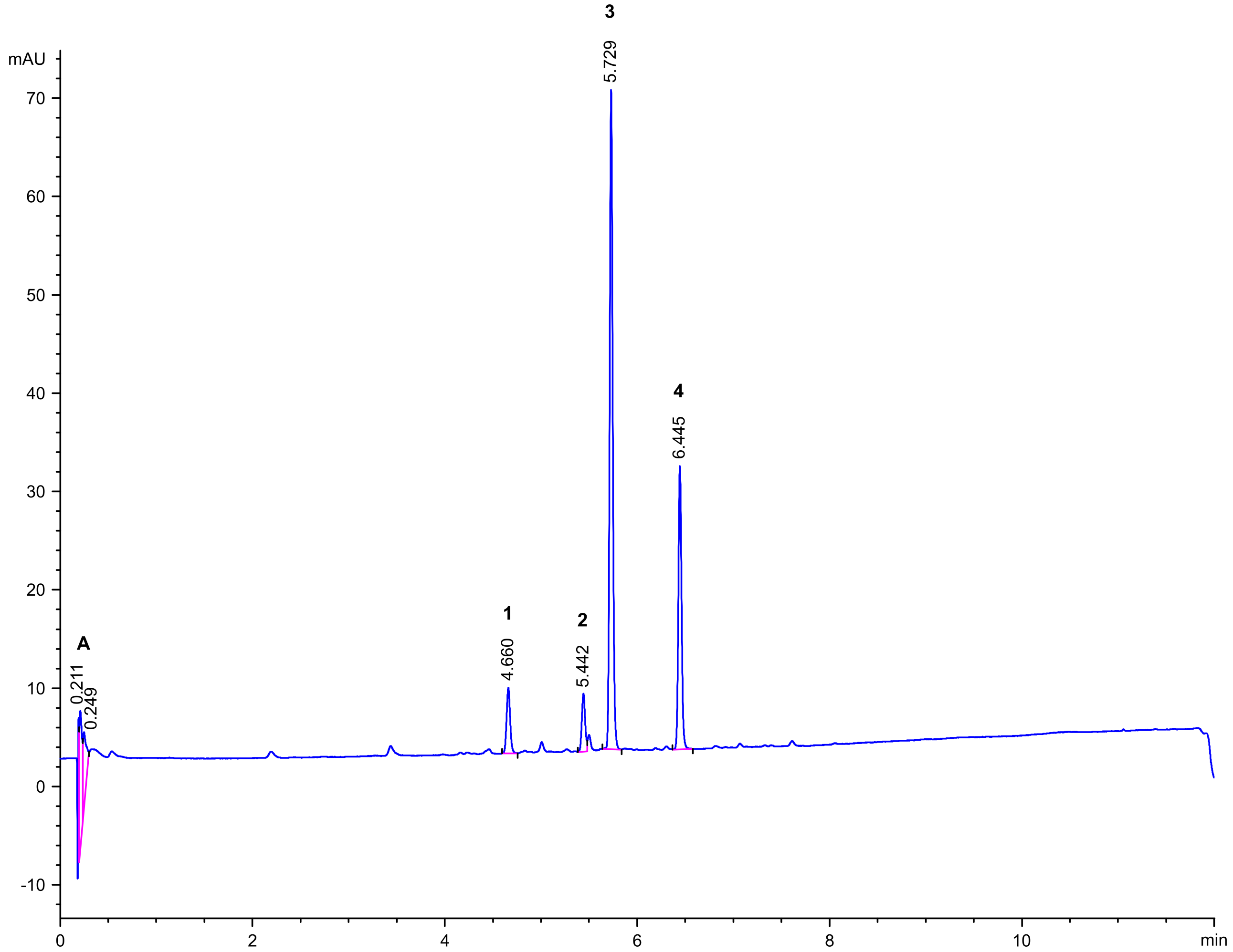


**Figure S5.** Chromatographic analysis (HPLC) of an acetone extract of *Umbilicaria crustulosa* (sample number 4128) at 254 nm. A = Acetone; 1 = Lecanoric acid; 2 = Unknown compound; 3 = Crustinic acid; 4 = Gyrophoric acid; mAU = milli-absorbance unit.

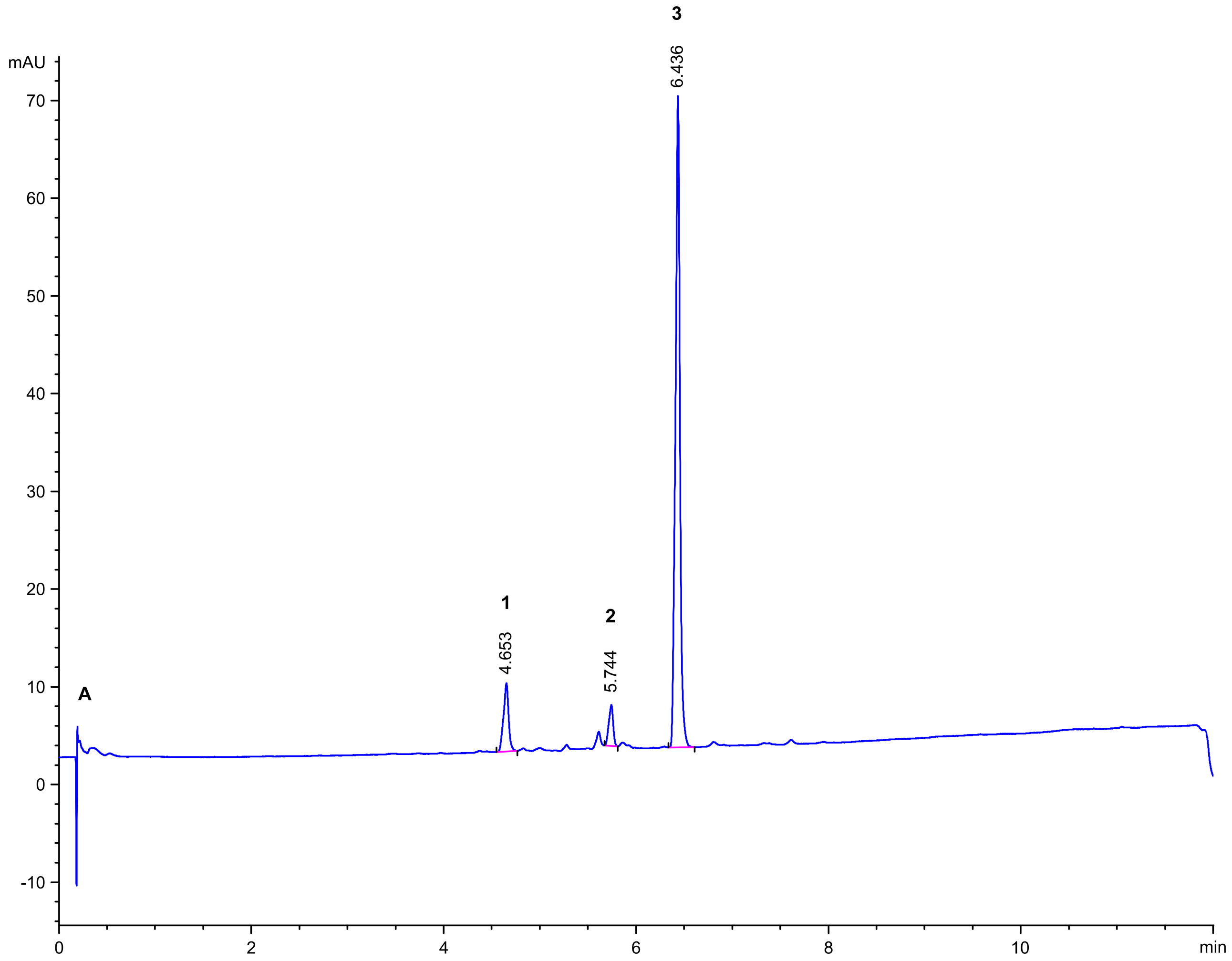


**Figure S6.** Chromatographic analysis (HPLC) of an acetone extract of *Umbilicaria pustulata* (sample number 4145) at 254 nm. A = Acetone; 1 = Lecanoric acid; 2 = Hiascic acid; 3 = Gyrophoric acid; mAU = milli-absorbance unit.
