# Supplementary material for: Unraveling the Pharmacological Potential of Lichen Extracts in the Context of Cancer and Inflammation With a Broad Screening Approach
Source: Front Pharmacol. 2020 Sep 4;11:1322. doi: 10.3389/fphar.2020.01322 (PMC7509413; doi:10.3389/fphar.2020.01322)
Supplement: Supplementary file 3 [file DataSheet_2.pdf]

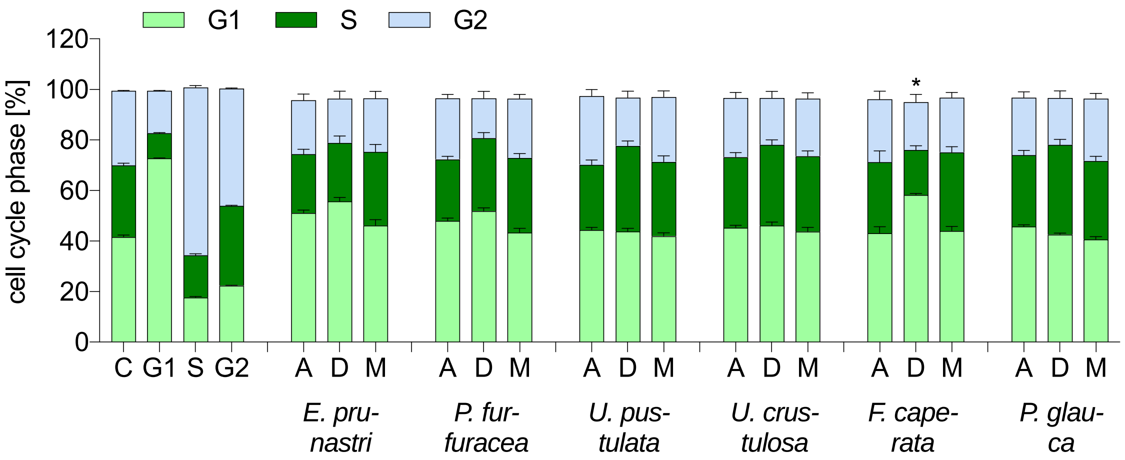


**Supplemental Figure 2**

For the cell cycle analysis HCT 116 cells were incubated with 3 µg/ml lichen extracts dissolved in organic solvent (A, D, M) over 24h. Cells were harvested, fixed with ice cold ethanol and stained with propidium iodide. Using flow cytometry the content of DNA (SSC channel) and the size of the cells (FSC channel) were determined. The analysis of the flow cytometry data was achieved with a specific cell cycle analysis method from the FlowJo software. Data are expressed as mean ± SEM. n=2, *$p \leq 0.05$ versus DMSO control.
