# Supplementary material for: Unraveling the Pharmacological Potential of Lichen Extracts in the Context of Cancer and Inflammation With a Broad Screening Approach
Source: Front Pharmacol. 2020 Sep 4;11:1322. doi: 10.3389/fphar.2020.01322 (PMC7509413; doi:10.3389/fphar.2020.01322)
Supplement: Supplementary file 4 [file DataSheet_3.pdf]

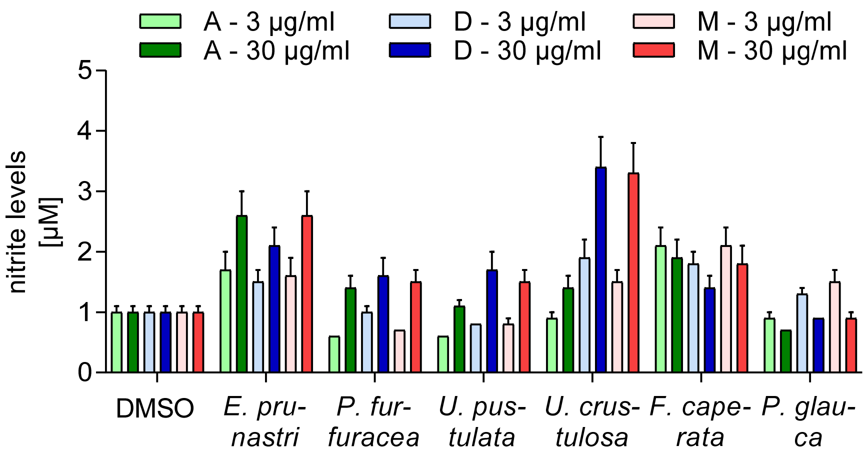

Supplemental Figure 3

For the NO assay, RAW macrophages were incubated with the indicated concentrations of lichen extracts dissolved in organic solvent (A, D, M) or DMSO (vehicle) over 24h. From the supernatant the concentration of NO was determined by colorimetric assay. Data are expressed as mean ± SEM. n=1.
