# Supplementary material for: Unraveling the Pharmacological Potential of Lichen Extracts in the Context of Cancer and Inflammation With a Broad Screening Approach
Source: Front Pharmacol. 2020 Sep 4;11:1322. doi: 10.3389/fphar.2020.01322 (PMC7509413; doi:10.3389/fphar.2020.01322)
Supplement: Supplementary file 5 [file DataSheet_4.pdf]

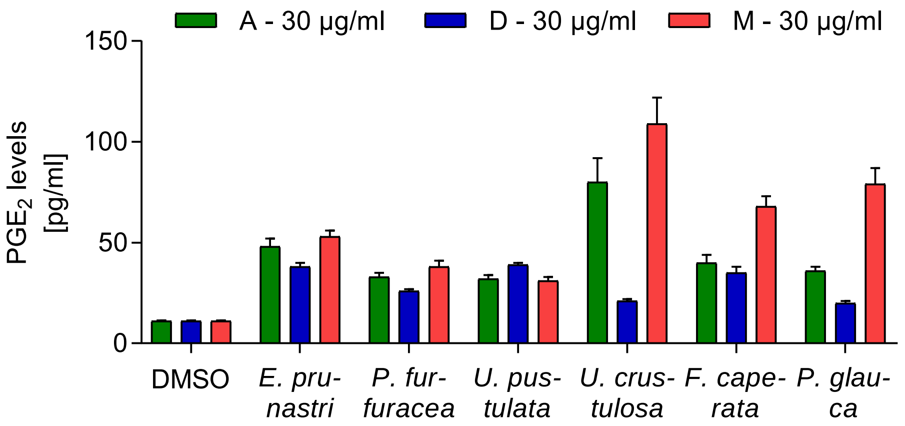

Supplemental Figure 4

For the PGE_2 assay, RAW macrophages were incubated with the indicated concentrations of lichen extracts dissolved in organic solvent (A, D, M) or DMSO (vehicle) over 24h. From the supernatant the concentration of PGE_2 was determined by ELISA. Data are expressed as mean ± SEM. n=1.
