# Supplementary material for: Unraveling the Pharmacological Potential of Lichen Extracts in the Context of Cancer and Inflammation With a Broad Screening Approach
Source: Front Pharmacol. 2020 Sep 4;11:1322. doi: 10.3389/fphar.2020.01322 (PMC7509413; doi:10.3389/fphar.2020.01322)
Supplement: Supplementary file 6 [file DataSheet_5.pdf]

			RT [min]					2.92	3.55	4.26	4.44	4.62	4.65	4.86	5.44	5.73	5.74	6.09	6.19	6.44	6.57	6.68	7.06	7.33	7.24	7.61	7.64	7.86	8.05	8.47
			**Compound**	(Solvent peaks)				Conproto-cetraric acid	Unidentified A	Confumarproto-cetraric acid	Norstictic acid	Protocetraric acid	Lecanoric acid	Unidentified B	Methyl lecanorate	Crustinic acid	Hiascic acid	Evernic acid	Oxyphysodic acid	Gyrophoric acid	Unidentified C	2'-O-Methyl-physodic acid	Physodic acid	Usnic acid	Alectoronic acid	Atranorin	Unidentified D	Unidentified E	Chloroatranorin	(Cyclo)aliphatic compound
**Solvent**	**Species**	**Sample name**		**Peak at 254nm**																										
Aceton	*Evernia prunastri*	3108	RT [min]	0.194														6.090						7.328		7.611				
			Area	13.9434									trace					566.5566						17.7826		13.7535			trace	
			Area%	2.2782														92.5691						2.9055		2.2472				
Methanol (70 %)	*Evernia prunastri*	3108	RT [min]	0.192														6.094						7.332		7.614			8.053	
			Area	25.4012					trace				trace					960.0634						34.1814		19.8825			11.3961	
			Area%	2.4170														91.3542						3.2525		1.8919			1.0844	
Dichlormethan	*Evernia prunastri*	3108	RT [min]	0.193														6.095						7.334		7.617			8.055	
			Area	10.5366														218.9583						14.6849		16.7933			13.1352	
			Area%	3.8440														79.8802						5.3573		6.1265			4.7920	
Aceton	*Evernia prunastri*	3121	RT [min]	0.194														6.090						7.328						
			Area	10.7380									trace					341.9404						12.4194		trace			trace	
			Area%	2.9411														93.6572						3.4017						
Methanol (70 %)	*Evernia prunastri*	3121	RT [min]	0.193														6.094						7.334		7.616			8.056	
			Area	33.7430					trace				trace					914.4638						24.2880		14.7786			12.4119	
			Area%	3.3754														91.4752						2.4296		1.4783			1.2416	
Dichlormethan	*Evernia prunastri*	3121	RT [min]	0.193														6.094						7.332		7.616			8.055	
			Area	10.6437														132.1838						14.3700		10.9893			12.2718	
			Area%	5.8981														73.2488						7.9631		6.0896			6.8004	
Aceton	*Pseudevernia furfuracea*	3325	RT [min]	0.193															6.194			6.682	7.061		7.241	7.610				
			Area	10.6544															98.4444			40.0271	344.0916		11.6890	14.0352			trace	
			Area%	2.0531															18.9702			7.7132	66.3064		2.2525	2.7046				
Methanol (70 %)	*Pseudevernia furfuracea*	3325	RT [min]	0.192															6.194			6.683	7.062		7.245	7.614				
			Area	27.7128					trace										163.9617			102.1916	667.5917		20.0799	11.1567			trace	
			Area%	2.7917															16.5168			10.2944	67.2505		2.0228	1.1239				
Dichlormethan	*Pseudevernia furfuracea*	3325	RT [min]	0.193															6.198			6.686	7.069			7.616			8.055	
			Area	10.8647															15.4648			53.5006	74.8095			21.8740			10.4164	
			Area%	5.8122															8.2730			28.6207	40.0201			11.7017			5.5723	
Aceton	*Pseudevernia furfuracea*	3332	RT [min]	0.193															6.190			6.678	7.056		7.241	7.611			8.051	
			Area	41.2920															193.2565			18.6481	930.3431		27.4971	28.5426			11.2119	
			Area%	3.3013															15.4507			1.4909	74.3804		2.1984	2.2820			0.8964	
Methanol (70 %)	*Pseudevernia furfuracea*	3332	RT [min]	0.192					3.554										6.196		6.567	6.718	7.066		7.247	7.614				
			Area	37.4947					13.1658										146.9494		11.5833	27.4513	642.8176		15.7591	12.6120			trace	
			Area%	4.1301					1.4502										16.1868		1.2759	3.0238	70.8079		1.7359	1.3892				
Dichlormethan	*Pseudevernia furfuracea*	3332	RT [min]	0.193															6.196			6.718	7.068			7.615			8.055	
			Area	10.3816															10.5288			11.5028	58.5227			32.8825			10.3018	
			Area%	7.7405															7.8502			8.5765	43.6345			24.5172			7.6810	
Aceton	*Umbilicaria crustulosa*	4128	RT [min]	0.211	0.249								4.660		5.442	5.729				6.445										
			Area	26.5978	16.9045								17.2343		14.0235	147.7337				66.9841										
			Area%	9.1882	5.8397								5.9536		4.8444	51.0345				23.1396										
Methanol (70 %)	*Umbilicaria crustulosa*	4128	RT [min]	0.194	0.224	0.262							4.668		5.447	5.729				6.445										
			Area	25.2648	21.4487	21.9176							19.5596		73.9351	92.5623				138.5090										
			Area%	6.4255	5.4550	5.5742							4.9745		18.8036	23.5410				35.2264										
Dichlormethan	*Umbilicaria crustulosa*	4128	RT [min]	0.192																										
			Area	12.0070																										
			Area%	100.0000																										
Aceton	*Umbilicaria crustulosa*	4129	RT [min]	0.211		0.252									5.439	5.723				6.438										
			Area	39.4456		14.7409							trace		18.5755	156.4866				81.0604										
			Area%	12.7117		4.7504									5.9861	50.4293				26.1225										
Methanol (70 %)	*Umbilicaria crustulosa*	4129	RT [min]										4.666		5.449	5.733				6.445										
			Area										13.0706		58.3101	57.6449				92.3372										
			Area%										5.9046		26.3414	26.0409				41.7131										
Dichlormethan	*Umbilicaria crustulosa*	4129	RT [min]	0.193																										
			Area	10.7858																										
			Area%	100.0000																										
Aceton	*Flavoparmelia caperata*	3636	RT [min]	0.193				trace		4.264		4.624												7.330						
			Area	11.4835						16.9301		269.7863												34.7511						
			Area%	3.4490						5.0849		81.0288												10.4373						
Methanol (70 %)	*Flavoparmelia caperata*	3636	RT [min]	0.192				2.959		4.275		4.633												7.333			7.637			
			Area	36.7041				14.5504		13.3781		748.6348		trace										51.9217			12.9358			
			Area%	4.1798				1.6570		1.5235		85.2538												5.9128			1.4731			
Dichlormethan	*Flavoparmelia caperata*	3636	RT [min]	0.193								4.627												7.334						
			Area	11.2677								15.9629												36.3530						
			Area%	17.7210								25.1054												57.1736						
Aceton	*Flavoparmelia caperata*	3656	RT [min]	0.194				2.941		4.271	4.435	4.629		4.865										7.334				7.860		
			Area	11.3606				12.1007		17.6646	23.2101	262.8320		13.3415										34.1452				11.8786		
			Area%	2.9391				3.1306		4.5700	6.0047	67.9973		3.4516										8.8337				3.0731		
Methanol (70 %)	*Flavoparmelia caperata*	3656	RT [min]	0.193								4.631												7.332		7.615				
			Area	11.7337				trace		trace	trace	282.9466												15.0252		30.5176				
			Area%	3.4488								83.1650												4.4163		8.9699				
Dichlormethan	*Flavoparmelia caperata*	3656	RT [min]	0.193								4.634												7.334		7.616		7.859		
			Area	10.3687							trace	10.0239												37.3601		10.0623		19.9659		
			Area%	11.8121								11.4192												42.5606		11.4629		22.7452		
Aceton	*Platismatia glauca*	4232	RT [min]	0.193																						7.613			8.052	
			Area	11.4660																						10.0903			12.4365	trace
			Area%	33.7307																						29.6836			36.5858	
Methanol (70 %)	*Platismatia glauca*	4232	RT [min]	0.193																									8.055	
			Area	17.4103					trace																	trace			10.0191	trace
			Area%	63.4733																									36.5267	
Dichlormethan	*Platismatia glauca*	4232	RT [min]	0.193																						7.617			8.055	
			Area	10.0030																						11.8433			10.2910	
			Area%	31.1258																						36.8523			32.0219	
Aceton	*Platismatia glauca*	4237	RT [min]	0.193																									8.053	
			Area	10.5759																						trace			12.0590	trace
			Area%	46.7238																									53.2762	
Methanol (70 %)	*Platismatia glauca*	4237	RT [min]	0.192																									8.054	
			Area	15.8003																						trace			10.7066	trace
			Area%	59.6081																									40.3919	
Dichlormethan	*Platismatia glauca*	4237	RT [min]	0.193																									8.058	
			Area	10.2566																						trace			10.7991	
			Area%	48.7117																									51.2883	
Aceton	*Umbilicaria pustulata*	4145	RT [min]										4.653				5.744			6.436										
			Area										25.9713				13.2225			217.2629										
			Area%										10.1270				5.1558			84.7172										
Methanol (70 %)	*Umbilicaria pustulata*	4145	RT [min]	0.193			0.493						4.648																	
			Area	47.4427			129.8286						33.4119							trace										
			Area%	22.5185			61.6227						15.8588																	
Dichlormethan	*Umbilicaria pustulata*	4145	RT [min]	0.193																										
			Area	11.8162																										
			Area%	100.0000																										
Aceton	*Umbilicaria pustulata*	4183	RT [min]	0.192									4.641							6.434										
			Area	47.4811									19.7518				trace			121.8796										
			Area%	25.1073									10.4445							64.4482										
Methanol (70 %)	*Umbilicaria pustulata*	4183	RT [min]	0.192	0.228		0.532						4.649							6.435										
			Area	21.5067	28.1497		65.5456						20.3601							10.0250										
			Area%	14.7724	19.3353		45.0216						13.9849							6.8859										
Dichlormethan	*Umbilicaria pustulata*	4183	RT [min]	0.193																										
			Area	11.5867																										
			Area%	100.0000																										
